# Soil-transmitted helminth infection, loss of education and cognitive impairment in school-aged children: A systematic review and meta-analysis

**DOI:** 10.1371/journal.pntd.0005523

**Published:** 2018-01-12

**Authors:** Noel Pabalan, Eloisa Singian, Lani Tabangay, Hamdi Jarjanazi, Michael J. Boivin, Amara E. Ezeamama

**Affiliations:** 1 Center for Research and Development, Angeles University Foundation, Angeles City, Philippines; 2 Department of Medical Technology, College of Allied Medical Professions, Angeles University Foundation, Angeles, Philippines; 3 Department of Biological Sciences, Angeles University Foundation, Angeles City, Philippines; 4 Environmental Monitoring and Reporting Branch, Biomonitoring Unit, Ontario Ministry of the Environment and Climate Change, Toronto, Ontario, Canada; 5 Department of Psychiatry, College of Osteopathic Medicine, Michigan State University, East Lansing, Michigan, United States of America; Texas A&M University College Station, UNITED STATES

## Abstract

**Background:**

Evidence of an adverse influence of soil transmitted helminth (*STH*) infections on cognitive function and educational loss is equivocal. Prior meta-analyses have focused on randomized controlled trials only and have not sufficiently explored the potential for disparate influence of *STH* infection by cognitive domain. We re-examine the hypothesis that *STH* infection is associated with cognitive deficit and educational loss using data from all primary epidemiologic studies published between 1992 and 2016.

**Methods:**

Medline, Biosis and Web of Science were searched for original studies published in the English language. Cognitive function was defined in four domains (learning, memory, reaction time and innate intelligence) and educational loss in two domains (attendance and scholastic achievement). Pooled effect across studies were calculated as standardized mean differences (SMD) to compare cognitive and educational measures for *STH* infected/non-dewormed children versus *STH* uninfected /dewormed children using Review Manager 5.3. Sub-group analyses were implemented by study design, risk of bias (ROB) and co-prevalence of *Schistosoma* species infection. Influential studies were excluded in sensitivity analysis to examine stability of pooled estimates.

**Findings:**

We included 36 studies of 12,920 children. *STH* infected/non-dewormed children had small to moderate deficits in three domains—learning, memory and intelligence (SMD: -0.44 to -0.27, P<0.01–0.03) compared to *STH*-uninfected/dewormed children. There were no differences by infection/treatment status for reaction time, school attendance and scholastic achievement (SMD: -0.26 to -0.16, P = 0.06–0.19). Heterogeneity of the pooled effects in all six domains was high (P<0.01; I^2^ = 66–99%). Application of outlier treatment reduced heterogeneity in learning domain (P = 0.12; I^2^ = 33%) and strengthened *STH*-related associations in all domains but intelligence (SMD: -0.20, P = 0.09). Results varied by study design and ROB. Among experimental intervention studies, there was no association between *STH* treatment and educational loss/performance in tests of memory, reaction time and innate intelligence (SMD: -0.27 to 0.17, P = 0.18–0.69). Infection-related deficits in learning persisted within design/ROB levels (SMD: -0.37 to -52, P<0.01) except for pre-vs post intervention design (n = 3 studies, SMD = -0.43, P = 0.47). Deficits in memory, reaction time and innate intelligence persisted within observational studies (SMD: -0.23 to -0.38, all P<0.01) and high ROB strata (SMD:-0.37 to -0.83, P = 0.07 to <0.01). Further, in S*chistosoma* infection co-prevalent settings, associations were generally stronger and statistically robust for *STH*-related deficits in learning, memory and reaction time tests(SMD:-0.36 to -0.55, P = 0.003–0.02). *STH*-related deficits in school attendance and scholastic achievement was noted in low (SMD:-0.57, P = 0.05) and high ROB strata respectively.

**Interpretation:**

We provide evidence of superior performance in five of six educational and cognitive domains assessed for *STH* uninfected/dewormed versus *STH* infected/not-dewormed school-aged children from helminth endemic regions. Cautious interpretation is warranted due to high ROB in some of the primary literature and high between study variability in most domains. Notwithstanding, this synthesis provides empirical support for a cognitive and educational benefit of deworming. The benefit of deworming will be enhanced by strategically employing, integrated interventions. Thus, multi-pronged inter-sectoral strategies that holistically address the environmental and structural roots of child cognitive impairment and educational loss in the developing world may be needed to fully realize the benefit of mass deworming programs.

## Author summary

Previous systematic reviews of the effect of *STH* infection on cognitive and educational performance were either inconclusive or found little to no evidence of associated benefit. Lack of consensus in prior reviews and their narrow emphasis on randomized controlled trials prompted this comprehensive assessment of whether educational and cognitive benefits are associated with the common practice of preventive chemotherapy among children in *STH-*endemic regions. This literature synthesis included all relevant epidemiologic studies regardless of design and investigated associations between *STH* infections and two outcomes: i) educational loss (attendance and scholastic achievement) and ii) psychometrically performance in four neurocognitive domains—memory, learning, reaction time and intelligence. Pooled results across 36 observational and intervention studies suggest that *STH* infection/non-treatment is associated with deficits in five of six domains evaluated and provide evidence in support of a cognitive and educational benefit of deworming for *STH* infections. This overall finding was sensitive to study design and risk of bias across studies.

## Introduction

Soil-transmitted helminthiases (*STH*) include infections with roundworm (*Ascaris lumbricoides*), whipworm (*Trichuris trichiura*) and hookworm (*Ancylostoma duodenale* and *Necator americanus*). *STH* infection affects one third of the world’s population [1, 2]. Primarily due to poverty, poor personal hygiene, frequent outdoor exposures, a higher likelihood of indulging in high risk behavior such as soil-eating, and the presence of environmental conditions favoring transmission [3, 4], school-age children between the ages of 5 to 15 in mostly developing countries are at highest risk of chronic helminth infection and helminth-associated morbidities [2, 4, 5]. Of note, the designation of school-aged children as highest risk may be because there has been insufficient research of the neurodevelopmental and cognitive importance of *STH* in toddlers [6–8] and preschool children [6, 9, 10]. In this highly affected demographic, *STH*-related morbidities occur during critical periods of physiologic, mental and physical development. The chronicity of infection ensures that any *STH*-related small to moderate nutritional, growth and cognitive deficits is cumulated over extended periods of the developmental life course.

Epidemiologic studies have reported lower health outcomes including- a higher prevalence of lethargy [11], stunting, wasting and anemia [12, 13] for helminth–infected relative to uninfected school-age children. In addition, some epidemiologic studies have reported a higher prevalence of school-absenteeism [13], and lower performance on a range of cognitive tests [14]. The mechanisms of adverse effect on cognition and mental function of infected children are not well understood [15] but helminth-associated iron-deficiency is thought to be important [1, 16, 17]. Recent evidence suggests that helminth-associated morbidity and mortality is likely magnified for polyparasitized children [18–20] and those with chronic untreated moderate and heavy intensity of infections.

Recent systematic reviews and meta-analysis of the health benefits of *STH* treatment based on intervention study designs concluded that the evidence for improvements in cognitive function was mixed and inconclusive and based on at best low quality evidence [15, 21]. Conclusions from these meta-analyses raise fundamental questions about the expected educational and cognitive benefits that partly justify current deworming programs in helminth endemic regions. However, the following criticism have been made of their conclusions [22–26]: (i) existing trials included were of poor quality [25]; (ii) interpretation did not factor in realities such as the high frequency and rapidity of reinfection following treatment [22]; (iii) and the fact that by offering one time treatment combined with the short-term duration of most trials, they have limited ability to evaluate long-term benefits of regular deworming as envisioned and supported by the World Health Organization [5], Unesco and the World Bank [23]. Proper assessment of long-term cognitive outcomes may require a sustained period of intervention and possibly post-treatment educational remediation in school-aged children [27–32].

Given the limitations of randomized trials noted above and a health policy environment that supports periodic deworming of school-aged children for expected growth, nutritional and perhaps cognitive benefits, clinical equipoise for unknown benefit of deworming is difficult to justify and the ethical landscape has coalesced around the understanding that randomizing children to deworming compared to placebo is largely unjust. Systematic reviews and meta-analyses remain an important tool for clarifying the possible cognitive impact of deworming. Including observational and intervention study designs in this synthesis provides robust scientific evidence to inform existing empirical gap in a way that enhances external validity beyond highly selected trial participants and conforms with the prevailing clinical, ethical and health policy environments that converge and contend with one another on the subject of deworming.

Hence, we incorporate all available epidemiologic evidence and re-examine the hypothesis that helminth infections adversely affect child cognitive function and educational outcomes. Unlike prior reviews, we examine the impact of *STH* infection on specific cognitive domains–learning, memory, attention/reaction time and intelligence, and do not assume equal impact of *STH* on across domains. We specifically hypothesize that *STH* infections will be associated with educational deficits and lower performance in learning, memory and attentional processes because these domains of child function are more sensitive to environmental assaults. We define a fourth domain “innate intelligence” that we expect to be mostly genetically determined and possibly less affected by *STH* infection.

## Methods

### Selection of studies

We performed a comprehensive search of databases including MEDLINE using PubMed (http://www.ncbi.nlm.nih.gov), Science Direct and Google Scholar, as of April 22, 2016 unrestricted by language. Throughout the data search, we used the key words, “*soil-transmitted helminthiasis*” and “*cognitive”* as well as "*intestinal parasites*", "*school performance*" and "*school attendance*". We included all studies–regardless of study design that had raw data for relationships between any of the 3 *STH* parasite species and educational indices (attendance or achievement) or neurocognitive outcomes. Articles were excluded if: (i) they were duplicates; (ii) completely lacked or incompletely presented needed raw data for relationships between *STH* and respective outcomes. We searched the body of included studies and their reference sections [32–35] for possible additional inclusions to this meta-analysis not identified by keyword searches. Our search yielded 7,709 citations and after a series of exclusions (outcomes attributed to other than *STH*, absence of required data and presence of duplicate data), provided for the final pool of articles included in the meta-analysis which was [13, 17, 31, 36–68]. We focused on the more recent publications (from the year 1992 onwards) in the hope of minimizing the high level of heterogeneity across studies prior to 1992 as noted by Watkins and Pollitt. [35].

### Psychometrically assessed cognitive functions

These were categorized into four domains: (i) memory, (ii) reaction time, (iii) learning, and (iv) intelligence tests. Many studies used a suite of psychometric instruments to assess a single or multiple cognitive domains in enrolled children. The memory domain included tests of working (short-term) memory as well as those of long-term memory. Attention/reaction time tests were those that measured the ability of a child to sustain concentration on a particular object, action, or thought, including their capacity to manage competing demands in their environments. The learning/executive function domain included tests to evaluate children’s performance in goal-oriented behavior, particularly components that are important for scholastic advancement. Executive function included tests of cognitive processes that enable children to connect past experience with present action, and by so doing, engage in planning, organizing, strategizing, paying of attention to details, and to emotionally self-regulate, make necessary efforts to remember important details required for attainment of future goals.[69] We included in the ‘intelligence’ domain psychometric tests of intelligence quotient (IQ) that we believe measures largely biologically determined cognitive abilities, in contrast to cognitive performance measures that are environmentally pliable.[70]

When multiple instruments were used to measure the same cognitive domain, a grand mean of scores and a grand mean of standard deviation (SD) across all instruments were calculated. Thus, for each publication, one overall mean and SD value was determined for each domain. A study could contribute data to different cognitive domains if it used tools spanning across several cognitive domains; however, each instrument only contributed to one single domain of function (S1 Table). Overall effects were derived only if there were two or more studies in a given domain.

### Educational deficits

Attendance rate was defined as the number of days children attended school over the past month (in cross-sectional studies) or over the study period (in longitudinal studies). In case-control studies, the percentage of children enrolled versus not enrolled in school was calculated for *STH*-infected and non-infected children.

The definition of scholastic achievement varied across studies. Children with high versus low attainment were identified based on: i) pass rate on standardized teacher-made tests, ii) percent of children that were in appropriate class for their age, iii) the enrollment of children in elite versus non-elite schools, iv) scores in the school function domain of pediatric quality-of-life inventory, v) change in class position after treatment for *STH* infection, vi) an above versus below average class performance as rated by a teacher, or vii) pass rate in any kind of educational test, whether teacher administered or not. (S1 Table).

### *STH* infection

*STH* infection status was determined by microscopic examination of stool. Operationally, infection was defined based on study design as follows: 1) untreated/placebo versus Albendazole / Mebendazole / Dicaris-treated in a randomized controlled trial (RCT), 2) any versus no *STH* infection in cross-sectional studies and 3) pre-versus post-Albendazole / Mebendazole / Dicaris treatment or infection-free versus persistent infection among *STH*-infected individuals in a longitudinal design study.

### Data extraction

Two investigators (NP, AEE) independently extracted data. Disagreements (if any) were resolved by a third person (LT). If resolution was not attained by the third author alone, abstracted information was resolved by consensus between LT, NP and AEE. The following information was obtained from each publication: first author’s name, published year, country of origin, parasite(s) involved, age range of the subjects, effect outcome of the study, study design, study features as well as subject features and sample sizes.

### Quality assessment of the included studies

We followed the Preferred Reporting Items for Systematic Reviews and Meta-Analyses (PRISMA) and Meta-analysis Of Observational Studies in Epidemiology (MOOSE) guidelines in describing our findings and standard methodology [71–73]. We assessed study quality using the Cochrane Collaborations tool for assessing risk of bias in randomized trials.[74] To assess methodological quality of each observational study, we modified the Newcastle-Ottawa scale (NOS) [75, 76] using the star * system based on the following: (i) representativeness of infected sample or case/control selection (min = 0, max = 3*); (ii) comparability using known correlates of cognitive function/educational attainment (min = 0, max = 6*). Here, we accounted for the confounding effects of age (score = 1*), sex (score = 1*), nutritional (score = 2*) and socioeconomic (score = 2*) status in relation to *STH* infection and educational/ cognitive outcomes. (iii) Absence of bias in relation to outcome assessment in prospective cohort studies (min = 0, max = 3*) or exposure assessment in cross-sectional and case-control studies (min = 0, max = 3*). For each study, the initial raw quality score (max = 12*) was rescaled to match the scale of 9* and then classified as low, high or very high risk of bias per prior literature precedent [76].

### Statistical analysis

Data were analyzed using Review Manager 5.3 (Copenhagen: Nordic Cochrane Centre, Cochrane Collaboration, 2014) and SigmaStat 2.3 and SigmaPlot 11.0 (Systat Software, San Jose, CA). We sought the number of children on two levels: (i) those who took the cognitive tests and compared those who were infected with those who were not; and (ii) those monitored for school achievement and attendance. We expressed these relationships as standardized mean difference (SMD) and 95% confidence intervals (CI). SMD estimates were classified as statistically significantly different if their confidence intervals did not cross zero. Most the included articles (studies) presented multiple means and measures of spread (SD or standard error (SE)). From each article, we obtained the mean of the multiple means and SDs which we used as input in generating forest plots. However, Some papers presented median (m) and range (a and b). These measures were converted into approximate mean and SD following Hozo et al [77]. Where mean and 95% CI or SE were reported, SD were derived as described by the Cochrane Collaboration [78]. For studies presenting data on differences in mean scores between two time points for treated/infected versus untreated/uninfected groups, appropriate SD for mean difference was calculated per the approach described by the Cochrane Collaboration [78]. Pooled SMDs were obtained using two analysis models based on presence or absence of heterogeneity: the fixed [79] and random [80] effects, respectively. Heterogeneity refers to diversity which may influence the manner in which the data are treated [81]. Significance was set at a P-value of <0.05. We addressed heterogeneity between studies with the following: (i) estimated using the χ^2^-based Q test [82]; (ii) its sources identified using the Galbraith plot method [83] and (iii) explored using subgroup analysis [82] where we examined effects in observational and interventional studies. Intervention studies, often prospective, are specifically tailored to evaluate direct impacts of treatment or preventive measures on disease. Observational studies on the other hand are often retrospective and used to assess potential causation in exposure-outcome relationships and therefore influence preventive methods [84]. In addition, we also examined effects according to risk of bias (low and high), all trial and pre-post studies as well *Schistosomiasis* coinfection.

Influence of each study on robustness of the summary effects and heterogeneity was determined with sensitivity analysis, which involved omitting one study at a time followed by recalculation of the pooled SMD. Change in direction of association (e.g. poor performance to better performance and vice-versa) after study omission indicates non-robustness of the summary effect, otherwise the pooled SMD is considered robust, indicating stability of the results. We investigated publication bias in domains with ≥ 10 studies because with < 10, sensitivity of the qualitative and quantitative tests of comparisons studies was low [85]. We implemented sensitivity analysis based on observational or experimental study design. Available data from studies that provided baseline treatment of children and post-test readings of the same subjects were included as intervention studies.

## Results

### Included studies

**Fig 1:**
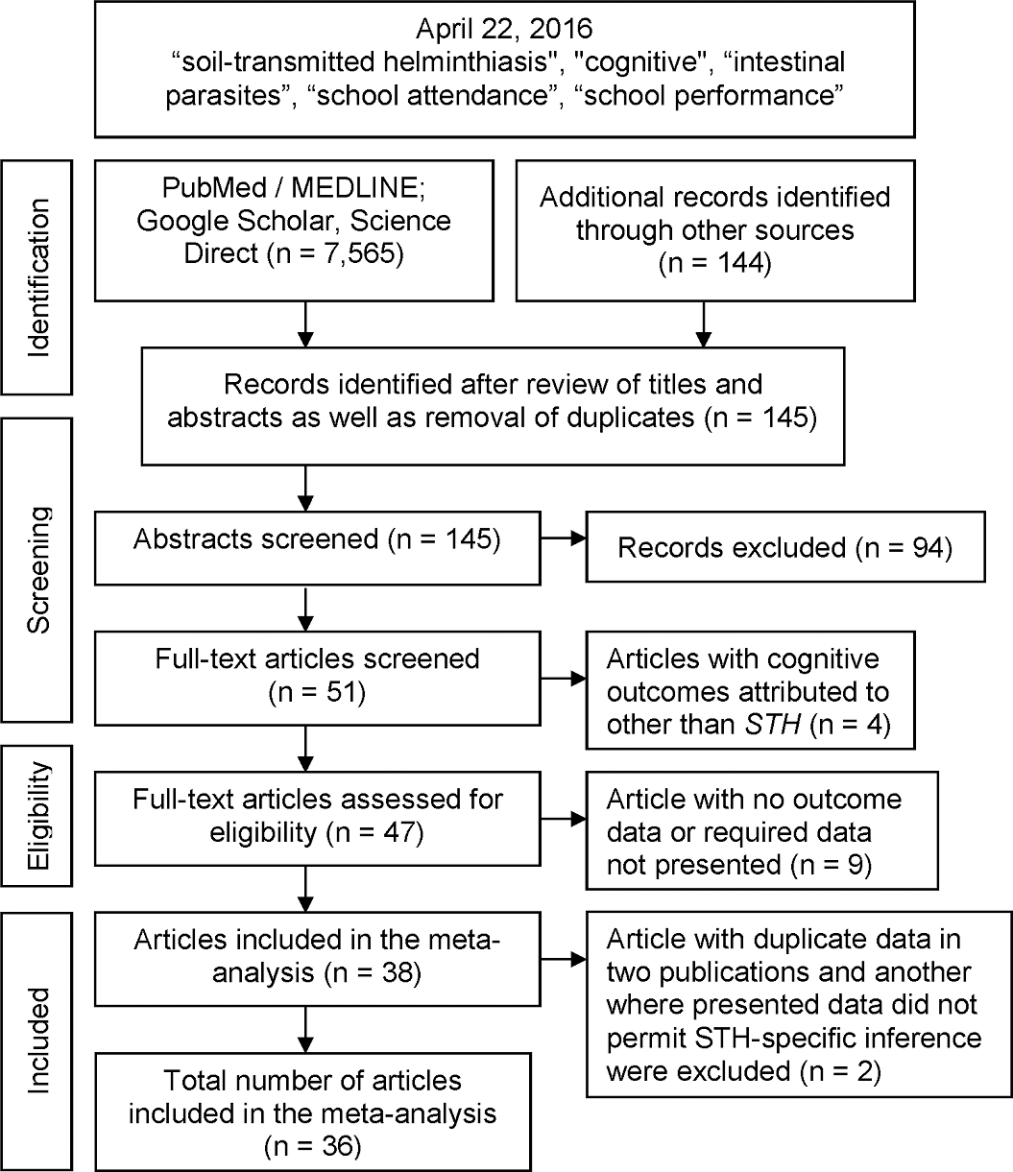
Summary flowchart of literature search.

We included 36 studies (Fig 1) of 12,920 children (5-20y) that evaluated *STH* associated differences in psychometrically evaluated cognitive tests, educational attainment or school attendance (Table 1). Of these, 5,932 children were evaluated in the context of *STH* treatment (with or without randomization) and 6,978 were evaluated as part of observational study. In the latter, 5,538 and 586 children were part of cross-sectional and longitudinal studies, respectively. The remaining 854 subjects were part of comparative Epidemiologic surveys. The PRISMA checklist is included with further pertinent details for this meta-analysis (S3 Table).

**Table 1:** Characteristics of the included studies.

	Author Year [reference]	Country	Parasite	Subjects age range	Effect on	Study design	Study features	Subjects and sample sizes
1	Jardim-Botelho 2008 [52]	Brazil	*Ascaris*, *Hookworm*	6-11y	Cognitive performance	O	Cross-sectional	Uninfected compared with infected either with hookworm or *Ascaris*. Sample sizes varied with these parameters.
2	Levav 1995 [54]	Ecuador	*Ascaris*, *Trichiura*	9-13y	Attendance	O	Baseline analysis of an intervention study	Subjects grouped according to level of nutrition and EEG results (normal/abnormal); Non-infected (K = 27) compared with infected (K = 66).
3	Lobato 2012 [56]	Brazil	*STH*	6-10y	Cognitive ability	O	Longitudinal with intervention	Infected and uninfected children compared in terms of cognitive tests. Those infected (n = 26) were then treated and allocated to educational (n = 15) vs. no educational intervention (n = 11). No adjustment for covariates via multivariable analysis. Very small sample size. Follow-up duration unclear.
4	Callender 1998 [42]	Jamaica	*Trichuris*	6.6–10.7y	Cognitive ability	O	Case-control	Follow-up study is reported of 18 children 4 year after treatment for the *Trichuris* dysentery syndrome (TDS) and matched control children.
5	Hutchinson 1997 [51]	Jamaica	*STH*	9-13y	School achievement and attendance	O	Cross-sectional	Up to 800 children were randomly selected from 16 different schools and examined for geohelminth infections related to school achievement and attendance.
6	Nokes 1992 [86]	Jamaica	*STH*	9-12y	Cognitive function	E	Random, double-blind	Randomized treated subjects (K = 62) compared with control (K = 56) and placebo (K = 41) subjects in baseline and repeat tests.
7	Nokes 1993 [13]	Jamaica	*Trichuris*	9-12y	Attendance	O	Cross-sectional survey	Attendance rates were compared for *Trichuris* infected and uninfected children.
8	Simeon 1995A [63]	Jamaica	*Trichuris*	6-12y	Spelling scores, Attendance	E	Random, double-blind	Separate data for baseline and post-test readings; placebo (K = 201) compared with treated (K = 206) groups.
9	Simeon 1995B [64]	Jamaica	*Trichuris*	7-10y	Cognition	E	Randomized clinical trial	Effects of *Trichuris* infection on cognitive functions were assessed in children randomly assigned to receive treatment (Albendazole) (K = 96) or a placebo (K = 93). Controls (K = 100) were included for comparison.
10	Simeon 1994 [62]	Jamaica	*Trichuris*	mean 9.1y	Achievement and Attendance	O	Cross-sectional	Examined relationship between varying intensities of *Trichuris* infection and school achievement and attendance in infected (K = 409) and uninfected (K = 207) children.
11	Gamboa 1998 [47]	Argentina	*STH*	<14y	Attendance	O	Cross-sectional	Prevalence of various intestinal parasite species examined in association with school attendance.
12	Gardner 1996 [48]	Jamaica	*Trichuris*	grades 2–5	Cognitive function	E	Random-controlled, double-blind treatment trial	97 subjects were randomly assigned to placebo (K = 48) or treatment (K = 49). Each pair of infected children was matched with an uninfected classmate (K = 48). Follow-up duration was 3 months. Intervention and cognitive battery was repeated at 3 months.
13	Sternberg 1997 [65]	Jamaica	*Trichuris*	10y	Cognitive function	E	Double-blind randomized controlled	Separate data for enrolment and post-test; treated (K = 66) compared with control (K = 63) and placebo (K = 67).
14	Theriault 2014 [66]	Peru	*STH*	5-14y	Attendance	E	Health hygiene education intervention, cluster-RCT study	Health hygiene education aimed at increasing knowledge of *STH* prevention were given to Grade 5 students (K = 517). Controls (K = 571) were Grade 5 students from the other nine schools.
15	Watkins 1996 [67]	Guatemala	*STH*	7-12y	Attendance	E	Random, double-blind	Week zero compared with week 24; placebo compared with treated groups; sample size varies with cognitive test used.
16	Bleakley 2007 [39]	USA	*Hookworm*	6-18y	Enrollment	O	Longitudinal	Retrospective assessment of impact of deworming for hookworm on area community educational enrollment. The sample consists of native-born whites and blacks 8–16 years old. Areas with pre-intervention high hookworm levels experienced largest increase in post-intervention enrollment, attendance and literacy.
17	Liu 2015 [55]	China	*STH*	9-11y	Cognitive ability	O	Cross-sectional	Large-scale survey of 2,179 children assessing the prevalence of *STH* infection in nationally-designated poverty counties in rural China.
18	Ziegelbauer 2010 [87]	China	*STH*	12-14y	Achievement	O	Cross-sectional	Assessed prevalence and intensity of *STH* infections and achievement in infected (K = 114) and uninfected (K = 138) schoolchildren.
19	Shang 2010 [61]	China	*STH*	9-12y	Cognitive structure	O	Cross-sectional	1,031 children were subjected to a battery of cognitive tests to assess their levels of cognitive abilities.
20	Ahmed 2012 [36]	Malaysia	*Ascaris Trichuris*	6-13y	Attendance	O	Pre-post intervention	This study assessed whether successful treatment of infection affected school attendance of the children (K = 289).
21	Ebenezer 2013 [43]	Sri Lanka	*STH*	Grade 4 (9y)	Cognitive abilities	E	Prospective, placebo-controlled randomized	Treatment group (K = 615) received deworming and weekly iron supplementation for 6 months; the control group (K = 575) received placebo for both the anthelmintic and iron.
22	Ezeamama 2005 [44]	Philippines	*STH*	7-18y	Cognitive impairment	O	Cross-sectional	Analysis of 319 children 6–18 years. Cognitive test scores for *STH (Ascaris and hookworm)* free (K = 68) subjects compared with those infected with any ascaris or hookworm (K = 215).
23	Ezeamama 2012 [45]	Philippines	*STH*	7-19y	Cognitive function	O	Longitudinal cohort	Examined changes in cognitive test scores over 18 months in relation to: (i) spontaneous reduction of single *STH* species, and (ii) *STH* infections among 253 *S*. *japonicum*-infected children.
24	Nga 2011 [57]	Vietnam	*STH*	6-8y	Cognitive outcome	E	Randomized, double-blind placebo controlled factorial trial	Assessed efficacy of multi-micronutrient fortified biscuits with or without de-worming on cognitive function in Vietnamese schoolchildren.
25	Hadidjaja 1996 [49]	Indonesia	*Ascaris*	6-8y	Cognitive function	E	Study using Mebendazole and health education as interventions	Mebendazole-treated and/or health education subjected children were compared with placebo individuals.
26	Sari 2012 [60]	Indonesia	*STH*	mean age of 9.6y	Cognitive function	E	Randomized, open-label, controlled trial	Compared Albendazole-treated (K = 56) and placebo groups (K = 57).
27	Sakti 1999 [59]	Indonesia	*Ascaris Trichuris*	8-13y	Cognitive function	O	Cross-sectional	Association between *STH* infection and cognitive/motor function was investigated in school-age children. 432 children from 42 primary schools participated in the study.
28	Beasley 2000 [37]	Tanzania	*STH*	7-12y	Attendance	O	Comparative study	Health of 227 children enrolled at primary school was compared with that of 214 non-enrolled children.
29	Berhe 2009 [38]	Ethiopia	*STH*	mean 13.4y	Attendance	O	Cross-sectional	For endemic and non-endemic areas, (K = 96) with *STH* and (K = 472) without *STH*.
30	Boivin 1993 [41]	Zaire	*Ascaris*	7-8y	Cognitive abilities	E	Controlled intervention	Positive (K = 47) or negative (K = 50) for intestinal parasites.
31	Fentiman 2001 [46]	Ghana	*Hookworm*	6-15y	Attendance	O	Comparative study	The study was designed to record simple indicators of health (particularly hookworm infected (K = 118) and non-infected (K = 100) and socio-economic status were recorded. Attitudes to enrolment among randomly selected children of school-age were explored.
32	Grigorenko 2006 [17]	Tanzania	*Hookworm*	≤11y	Cognitive skills over time	O	Cohort with Intervention	Three time periods, infected groups either treated (K = 270) or untreated (K = 447).
33	Hurlimann 2014 [50]	Cote d'Ivoire	*STH*	5-14y	Cognition	O	Pre-post Intervention	8–14 years, all children attending grades 4–6 in the two primary schools were invited to participate. Entire sample treated for infection (*STH* & Schistosomes) at enrollment and again 5 months later. Change in cognitive test score compared for *STH* Infected & uninfected.
34	Jukes 2002 [53]	Tanzania	*Hookworm*	9-14y	Cognitive function	O	Baseline cross-sectional study	Battery of 11 cognitive and three educational tests were given to schoolchildren and assessed for helminth infection.
35	Miguel and Kremer 2004 [31]	Kenya	*STH*	8-20y	Attendance & Achievement	O	Values directly obtained from publicly available data	We specifically compare school attendance and achievement of children persistently free of any *STH* infection at moderate to high intensity (K = 51) to attendance and achievement of children *STH* infected at moderate or high intensity in one or both follow-up years (K = 546).
36	Olsen 1998 [58]	Kenya	*Ascaris Trichuris*	6-15y	Attendance	O	Analyzes data from cross-sectional survey	Prevalence of *STH* infection in enrolled (K = 203) and non-enrolled (K = 53) school-aged children were examined.

*STH*: Soil-Transmitted Helminths; O: Observational; E: Experimental; K: number; y: year; EEG: Electroencephalogram

### Methodological quality and risk of bias

**Table 2** ranks included observational studies in terms of methodological quality using the NOS. Fourteen (56%) of these were determined to have low risk of bias and 11 studies (44%) were classified as having high risk or very high of bias. The ten intervention studies with featuring allocation of trial participants to deworming (alone or in combination) versus control groups were communicated across 11 publications. Seventy percent of trials had moderate or high risk of bias and thirty percent were uncertain to low risk of bias (**Table 3**).

**Table 2:** Risk of bias in observational studies using the Newcastle-Ottawa quality assessment scale.

First author & Year	Possible Coinfection with Schistosome species^ɤ^	Quality Assessment Notes	Selection max = 3	Comparability max = 6	Exposure / Outcome max = 3	Scaled Score max = 9	Risk of Bias
Lobato 2012	No	Infected and uninfected children were compared in terms of cognitive tests. There were no differences between these groups. Those infected (n = 26) were subsequently treated and allocated to educational (n = 15) vs. no educational intervention (n = 11). There was no adjustment for covariates via multivariable analysis. Very small sample size. Follow-up (FU) duration unclear.	*		*	2	Very high
Nokes 1993	No	This is a cross-sectional (CS) survey of 694 children from 3 schools in Jamaica. The goal was to determine the extent to which compliance with an *STH* screening protocol was related to infection intensity and school attendance. 90% of eligible children participated and 74% returned the second stool specimen. Only descriptive analyses. No adjustment for age, sex, socio-economic status (SES), nutritional status. School attendance was determined from school registers.	**		**	3	Very high
Olsen 1998	Yes	This is a CS survey including 256 children. Also included are preschool (<6 y) and adult (>15 y) groups. Abstraction restricted to 256; 6–15 yr old kids. 90% of eligible persons in village included. Parasitology via Kato-Katz (KK). School enrolment data via self-report.	*	*	**	3	Very high
Beasley 2000	Yes	Comparison of enrollment rate in infected and uninfected children. Mostly descriptive analysis presented but information on SES, nutritional status and other factors evaluated by infection status.	***		**	4	High
Callender 1998	No	Study of 18 children 4 years after *Trichuris* dysentery syndrome (TDS) and age, sex and neighborhood matched control children (n = 16). Standardized tools used for outcome assessment. Only descriptive analyses presented although matching might have controlled for age, sex and to some extent neighborhood level SES.	*	**	**	4	High
Gamboa 1998	No	Descriptive analysis of school attendance by *STH* infection. Major objective was comparison of *STH* prevalence by regions of marked SES differences. All analyses are descriptive. Very small sample size of uninfected controls. Infection assessed via KK but school attendance via self-report.	**	**	*	4	High
Shang 2010	No	Included 77 selected from over 1,000 children 9 to 12 years. Stool assessment via KK. Standardized assessment using psychometric test. No evidence of blinding to infection/ health status. Age, sex, SES measured. These three factors equally distributed by case/control status. No assessment of nutritional status. Descriptive analyses/results presented.	***	**	*	5	High
Ziegelbauer 2010	No	Survey of 252 students in 5–8 grade from China. Infection assessed via duplicate KK + single FLOTAC examination. Student end of term marks were obtained from teachers in select subjects—Chinese, English, Math. Multivariable analyses adjusted for age and sex only.	**	****	*	5	High
Bleakely 2007	Yes	Retrospective assessment of impact of deworming for hookworm on area community educational enrollment. The sample consists of native-born whites and blacks 8–16 years old. Areas with pre-intervention high hookworm levels experienced largest increase in post-intervention enrollment, attendance and literacy.	**	****	**	6	High
Fentiman 2011	Yes	Prevalence of *STH* infection in enrolled and unenrolled school aged boys matched for age and sex or class (if age-inappropriate for class). No evidence of multivariable analysis but no expected confounding by age, sex if goal of matching is realized.	***	***	**	6	High
Levav 1995	No	Sample of children enrolled for an intervention study. CS analysis of baseline data compared joint *STH* and protozoa infections (n = 66) vs. uninfected children (n = 27). The study had detailed measures of malnutrition, SES. Multivariable analysis clearly controlled for nutritional status. No evidence of multivariable control for SES, age or sex in analysis. Infection status measured via single KK. Outcome assessors blinded to child health and nutritional status.	**	****	**	6	High
Ahmed 2012	No	Sample included 254 of 364 children enrolled in a given school. FU duration = 3 months. Directly observed therapy for infected children at baseline. Detailed measurement of confounding covariates: age, sex, nutritional status, SES. These factors included in multivariable analyses.	**	*****	*	7	Low
Boivin 1993	No	Test retest study of 97 kids. Inferential interest on change in KABC post intervention. Cog assessment was blinded. Infection status determined by microscopy. Multivariate analyses controlled for nutrition, SES, but not age or sex.	***	****	**	7	Low
Jardim-Botelho 2008	Yes	196 children 6 to11 years attending one of 7 public elementary schools in Brazil. Parasitological assessment via KK. Multivariable control for a range of factors—age, sex, SES; but not nutritional status.	***	****	**	7	Low
Simeon 1994 S	No	616 children enrolled including with and without trichuris infection from Jamaica. Enrolled kids from schools and districts where infection prevalence was high. Controls were matched to cases on bases of classroom and age. Analytically controlled for age, sex, SES, nutritional status.	*	****	*	7	Low
Berhe 2009	Yes	Multivariable investigation of infection related differences in psychometric tests. Controlled analytically for several confounders including SES, nutritional status. Surrogate for attention "severe cramps distracting class attentiveness" has some measure of subjectivity.	**	******	**	8	Low
Ezeamama 2005	Yes	Analysis of 319 children 6–18 years. Used validated instruments. Controlled for: age, sex, hemoglobin status, nutritional status, SES & coincident *STH* infections	***	******	**	8	Low
Hurlimann 2014	Yes	All children were treated for both *STH* and schistosome infections at enrollment with repeat for both treatments at 5 months FU.	***	******	*	8	Low
Hutchinson 1997	No	Included only 5th graders from twenty-one primary schools in rural Jamaica. By design this study is age-stratified. Multi-variable regression models adjusted for age, sex, nutritional status, SES indicators and additional covariates.	***	******	**	8	Low
Jukes 2002	Yes	Large study of 338 uninfected, moderate or heavy schistosome infection with or without coinfection with moderate intensity hookworm. Objective outcome assessment including psychometric properties of used tests. Detailed confounder information including SES, nutritional indices, inflammation and malaria coinfections with multivariable adjustment.	***	******	**	8	Low
Liu 2015	No	Included 2179 children 9–11 years from seven nationally designated poverty counties in rural China. School attendance assessed via class room teacher. Scholastic achievement was measured via standardized testing. Infection was measured via KK. Multivariable analyses adjusted for age, sex, SES, nutritional status and other pertinent confounders beyond these ones.	***	*****	*	8	Low
Miguel and Kremer 2004	Yes	Prospective investigation of scholastic achievement and attendance by infection status over 24 months. Present study obtained attendance rate information by infection status from publicly available dataset. Children moderately/highly infected with *STH* in both years (n = 115) are compared with children free of moderate or high intensity *STH* infection in both years (n = 51).	***	******	**	8	Low
Ezeamama 2012	Yes	Treatment reinfection study among 253 schistosome infected children followed for 18 months with repeated assessment for infection and cognitive function with battery of psychometric tests. Analytically controlled for: age, sex, hemoglobin status, nutritional status, SES & coincident *STH* infections in evaluating association between infection free duration and performance in four cognitive tests	***	******	***	9	Low
Grigorenko 2006	Yes	Large cohort study with intervention for some children. Sample was randomized to screening vs. no screening for *STH* and *Schistosoma species*; 64 weeks of FU. Screened, infected were treated with Albendazole + Praziquantel. Uninfected among screened children were not treated. All randomized to no screening were untreated although a group of children that were infected but untreated in the non-screened arm were distinguished from those randomized to no screening but were uninfected and therefore would appropriately not need to be treated. Multivariable analyses contrasted performance for children infected & untreated (randomized to no screening arm), infected treated (randomized to screening arm) vs. children that were uninfected not treated status. Robust statistical control of multiple confounders.	***	******	***	9	Low
Sakti 1999	No	Included 432 children from 40+ schools. Infection assessed via KK; neurocognitive outcomes via standardized instruments. No blinding. Controlled for extensive matrix of confounders via multivariable analyses: age, sex, SES, nutrition, etc.	***	*	***	9	Low

ɤ: Sample is considered at risk of schistosome co-infection if schistosoma species are also measured and found to be present at any level. Data resolution does not allow for accurate separation of coinfected children.

**Table 3:** Risk of bias analysis for intervention studies using cochrane collaborations’ tool.

Study	Trial Assessment Notes	Random Sequence Generation	Allocation Concealment	Blinding of Participants & Personnel	Blinding of Outcome Assessment	Incomplete Outcome Data	Selective Reporting	Other Bias	Risk of Bias Judgement
Nga 2011	2x2 factorial design random allocation to neither, either or joint treatment with fortified biscuit and deworming. 4 months follow-up (FU) duration. High Retention.	+	+	+	+	+	?	+	Low
Watkins 1996	Randomized allocation to Albendazole vs. placebo. Repeated treatment at baseline and 12 weeks later. Implementation team blinded to child infection status & treatment arm.	+	?	+	+	+	+	+	Low
Gardner 1996	Placebo controlled trial with allocation to Albendazole vs placebo. Age matched group of uninfected classmates also enrolled. FU was 3 months. Retention rate was 97%.	?	?	+	+	+	+	+	Low to Uncertain
Ebenezer 2013	Albendazole + iron supplementation vs. neither treatment in 100 Sri Lankan schools. Intervention vs. control school clusters matched according to *STH* prevalence. Random allocation in block sizes of two with additional concealment measures. Outcome assessors were blinded to treatment status. By design assessing joint *STH* & iron effects.	+	+	+	+	-	?	-	Moderate
Nokes 1992	Double blind randomized controlled study of deworming vs. placebo. Uninfected control group included. 169 kids across all 3 groups. High retention.	+	+	+	?	?	?	+	Moderate
Sternberg 1997	Double blind placebo controlled study in 133 Jamaican children with moderate Trichuris infections sex matched to another classmate then randomized to either Albendazole or placebo. *STH* uninfected classmates included as control.	+	?	?	?	+	?	+	Moderate
Simeon 1995	Randomized allocation to Albendazole vs. placebo in 407 Jamaican children with moderate intensity trichuris infections. Participants were in grades 2–5 residing in Jamaica and attending 14 different schools in Jamaica. Follow-up duration was 6 months. Analyses examined a range of outcomes in different publications.	+	?	?	?	?	?	+	High
Sari 2012	Non-blinded randomized trial Albendazole vs. placebo in *STH* infected children. Table 1 of original publication suggests groups are similar with respect to age, sex, nutritional status, infection and background cognitive test scores. No measurement of socio-economic (SES) factors. High retention.	+	-	-	-	+	+	?	High
Theriault 2014	Dewormed children were cluster randomized within schools to *STH* focused hygiene education vs. usual education. Absenteeism rate was measured via school attendance logs. Multivariable analyses implemented with adjustment for age, sex, SES; coinfections but not nutritional status.	+	?	-	?	-	+	?	High
Hadidjaja 1996	2X2 Cluster randomized trial; allocation to single or joint Albendazole, education vs. respective placebo. Six months FU duration with ~25% attrition. At baseline infection levels and cognitive test scores were not comparable across intervention groups.	-	-	-	-	?	+	?	High

### *STH* species and treatment effects

S2 Table shows that the vast majority of included studies examined educational or cognitive impact infection with multiple *STH* species (up to 62.5%). *Ascaris* infections were the least studied (none in three domains). Nineteen studies (52.7%) included in this meta-analysis used Albendazole or Mebendazole for *STH* treatment with the exception of Boivin *et al* [41] where Decaris (Tetramisole, Lavamisole) was used.

### Impact of *STH* infection on educational loss and cognitive functions

**Fig 2:**
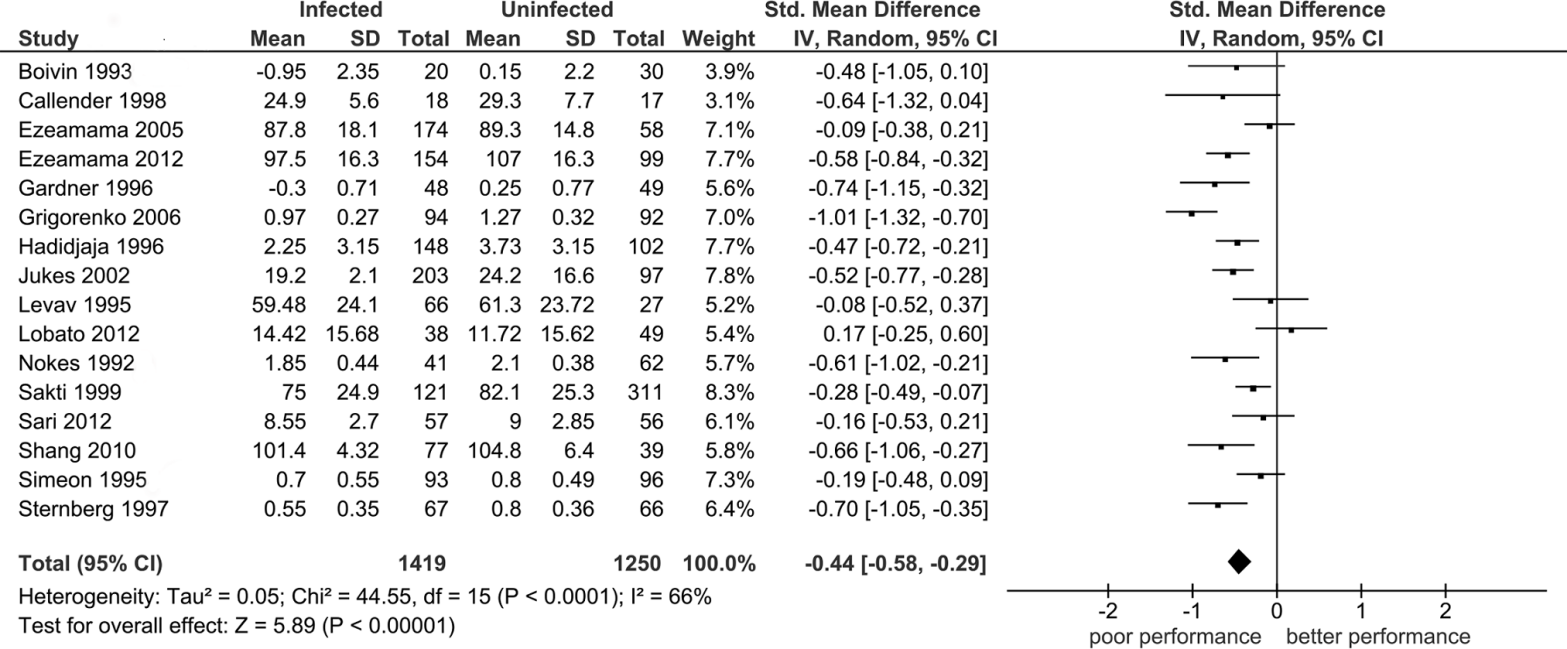
The association between soil-transmitted helminth infection or no-deworming on tests of learning. SD: standard deviation; CI: confidence interval; Std: standard; df: degree of freedom; I^2^: measure of variability expressed in %. Squares indicate the SMD in each study, with square sizes directly proportional to the weight contribution (%) of each study. Horizontal lines represent 95% confidence intervals (CI). The diamond denotes the pooled standardized mean difference (SMD). The Z test for overall effect indicates deficits for *STH* infected/non treated vs. uninfected/treated children. The chi-square test indicates heterogeneity is high (P <0.00001, I^2^ = 66%) warranting use of the random-effects model.

**Fig 3:**
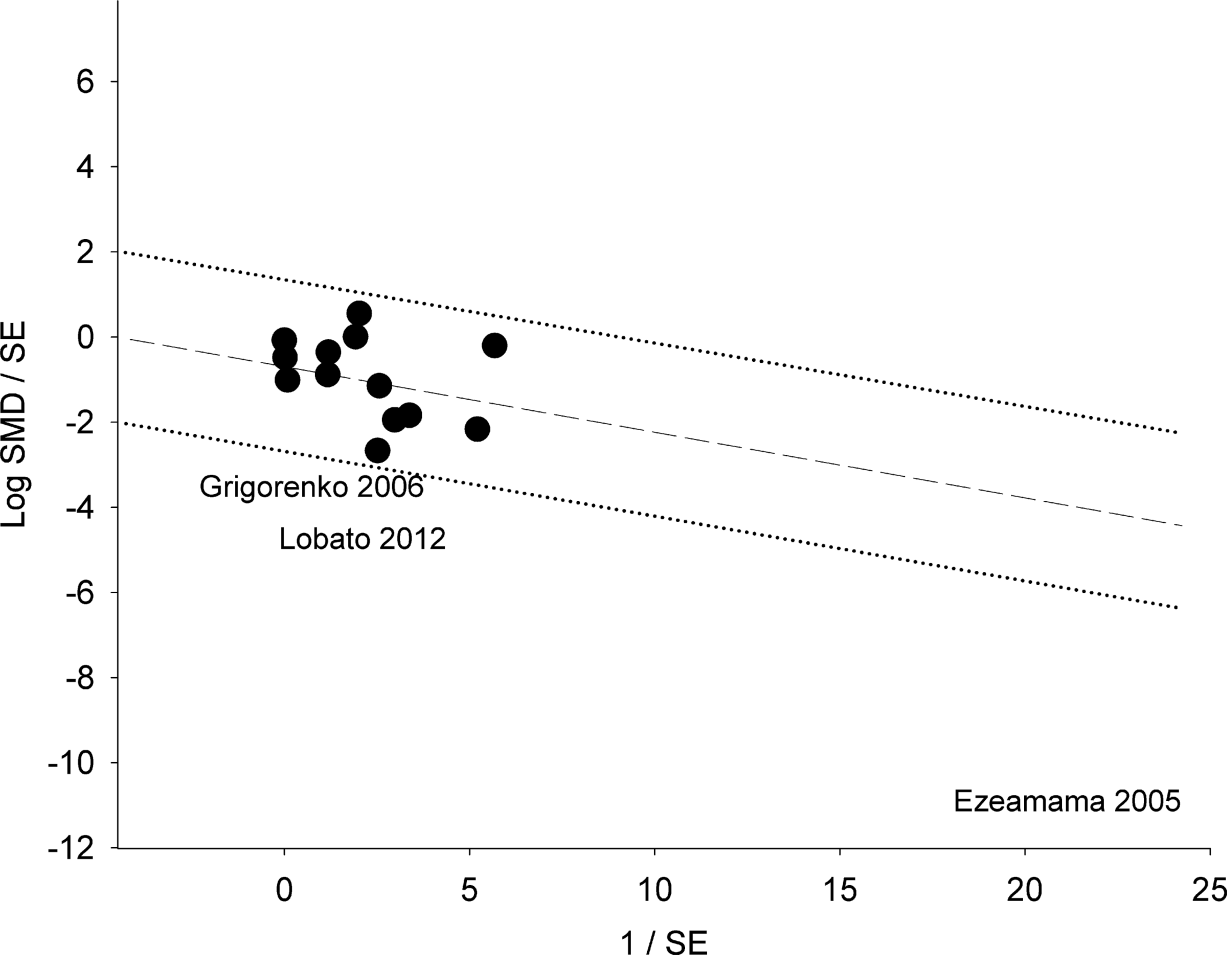
Galbraith plot analysis to identify outliers among included studies of *STH* infection and performance in learning tests. Log SMD: logarithm of standardized mean difference; SE: Standard error. Studies that lie below the– 2 or above the +2 confidence limit are the outliers.

**Fig 4:**
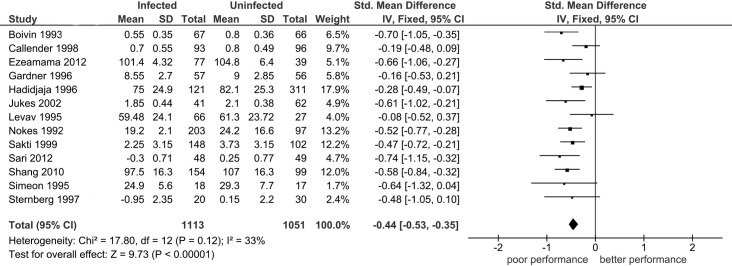
The association between soil-transmitted helminth infection or no deworming on tests of learning with exclusion of identified outlier investigations. Diamond denotes the pooled standardized mean difference (SMD). Squares indicate the SMD in each study, with square sizes directly proportional to the weight contribution (%) of each study. Horizontal lines represent 95% confidence intervals (CI). The Z test for overall effect indicates non-significance given that *P* > 0.05. The chi-square test indicates that heterogeneity (P = 0.12, I^2^ = 33%) was reduced by outlier treatment. SD: standard deviation; CI: confidence interval; Std: standard; df: degree of freedom; I^2^: measure of variability expressed in %.

As shown in Table 4, the overall analysis shows that *STH*-infected/non-treated children performed significantly worse than uninfected/dewormed children in the three of the six domains including: memory (SMD: -0.31, P = 0.01); learning (SMD: -0.44, P <0.0001, Fig 2) and intelligence (SMD: -0.27, P = 0.03). Because pooled effects in all six domains were obtained under highly heterogeneous conditions (P_heterogeneity_ <0.01, I^2^ = 66–99%), we identified investigations contributing to large variability in the pooled effect of *STH*-infection/non-treatment on performance in each of the cognitive and educational domains using the Galbraith plot technique (Fig 3). This technique did not significantly reduce heterogeneity in respective outcomes with the exception of the learning domain (I^2^ = 33%, Fig 4). However, application of this technique resulted in emergence of statistically robust estimates of effect in three domains: reaction time (SMD: -0.21, P = 0.004), achievement (SMD: -0.24, P <0.01) and attendance (SMD: -0.52, P <0.01). On the other hand, exclusion of one influential study (Shang et al [61]) resulted in loss of statistical significance in intelligence domain (SMD: -0.20, P = 0.09).

**Table 4:** Associations between *STH* infection/non-treatment and performance in cognitive and educational domains among school-aged children following exclusion of outlier investigations using the Galbraith plot method.

	Overall Findings							Outlier Analysis									
		Test of Association			Test of Heterogeneity					Test of Association			Test of Heterogeneity				
Domain	K	SMD	95% CI	P_A_	P_B_	I^2^ (%)	AM	*Studies omitted*	K	SMD	95% CI	P_A_	P_B_	I^2^ (%)	AM	K studies omitted	Effect of Outlier Treatment
Memory	20	**-0.31**	**[-0.55, -0.06]**	**0.01**	<0.001	94	R	[48, 86]	18	**-0.20**	**[-0.36, -0.04]**	**0.02**	<0.001	84	R	2	NEAH
Learning	16	**-0.44**	**[-0.58, -0.29]**	**<0.001**	<0.001	66	R	[17, 44, 56]	13	**-0.44**	**[-0.53, -0.35]**	**<0.001**	0.12	33	F	3	LOH, NEA
Reaction time	20	-0.18	[-0.38, 0.01]	0.06	<0.001	89	R	[41, 53, 86]	17	**-0.21**	**[-0.36, -0.07]**	**0.004**	<0.001	78	R	3	GIS, NEH
Intelligence	9	**-0.27**	**[-0.52, -0.03]**	**0.03**	<0.001	80	R	[61]	8	-0.20	[-0.44, 0.03]	0.09	<0.001	77	R	1	LOS, NEH
Achievement	16	-0.16	[-0.37, 0.04]	0.11	<0.001	93	R	[17]	15	**-0.24**	**[-0.41, -0.06]**	**0.008**	<0.001	90	R	1	GIS, NEH
Attendance	15	-0.26	[-0.64, 0.13]	0.19	<0.001	99	R	[67]	14	**-0.48**	**[-0.81, -0.15]**	**0.004**	<0.001	98	R	1	GIS, NEH

K: number of studies; SMD: standard mean difference; CI: confidence interval; P_A_: P-value for association; P_B_: P-value for heterogeneity; I^2^: measure of variability expressed in %; AM: analysis model; R: random effects; F: fixed-effects; Values in **bold** indicate significant associations; NEAH: no effect on association and heterogeneity; LOH: loss of heterogeneity; NEA: no effect on association; LOS: loss of significance; NEH: no effect on heterogeneity; GIS: gain in significance

### Subgroup analysis

Stability of overall findings were evaluated in sub-groups distinguished by study design, presence of non-outlier studies and study quality (Table 5). In the context of observational study designs, *STH* infection/non-treatment was significantly and consistently associated with deficits in memory, learning, reaction time and intelligence with SMD ranging from -0.42 to -0.23 (P <0.01–0.05). Among studies featuring STH treatment- pre-post longitudinal study or experimental in design, moderate (SMD: - 0.8, P = 0.14) to large (SMD: -0.46, P<0.0001) deficits in memory and learning tests were respectively noted. was evident for untreated children. With the exception of the learning domain (SMD = -0.40, P<0.0001), there was no association between non-treatment for *STH* and performance in any of the other five outcome domains for pooled estimates of experimental study designs only (SMD -0.27, 0.27, P = 0.18–0.69). Among observational studies featuring pre-post *STH* assessment of cognitive or educational outcomes, infection related deficits was evident in test of reaction time only (SMD -0.37, P<0.001) with corresponding substantial reduction in heterogeneity (P_heterogeneity_ = 0.72, I^2^ = 0%).

**Table 5:** Effect of *STH* infection/non-treatment on performance in cognitive and educational within strata of study designs, non-outlier investigations and study quality.

		Test of Association			Test of Heterogeneity		
	K	SMD	95% CI	P_A_	P_B_	I^2^ (%)	AM
Memory							
Observational	14	**-0.23**	**[-0.44, -0.03]**	**0.02**	<0.00001	87	R
All studies with *STH* treatment*	5	-0.80	[-1.87, 0.26]	0.14	<0.00001	98	R
Experimental intervention Studies	2	-0.03	[-0.17, -0.11]	0.69	0.85	0	F
Pre-post intervention studies	3	-0.11	[-1.05, 0.84]	0.82	<0.0001	96	R
Low risk of bias**	10	-0.07	[-0.34, 0.19]	0.59	<0.00001	93	R
High risk of bias***	8	-0.83	**[-1.50, 0.16]**	**0.07**	<0.00001	96	R
Learning							
Observational	10	**-0.42**	**[-0.63, -0.21]**	**0.0001**	<0.0001	74	R
All studies with *STH* treatment	8	**-0.47**	**[-0.72, -0.22]**	**<0.0001**	0.08	50	R
Experimental Intervention Studies	5	**-0.40**	**[-0.55, -0.25]**	**<0.0001**	0.85	0	F
Pre-post intervention studies	3	-0.43	[-0.59, 0.73]	0.47	<0.0001	95	F
Low risk of bias	7	**-0.52**	**[-0.75, -0.29]**	**<0.0001**	0.0005	75	R
High risk of bias	9	**-0.37**	**[-0.56, -0.17]**	**0.0002**	0.02	57	R
Reaction time							
Observational	12	**-0.38**	**[-0.60, -0.16]**	**0.0007**	<0.00001	90	R
All studies with *STH* treatment	**9**	0.01	[-0.24, 0.27]	0.91	<0.0001	87	R
Experimental Intervention Studies	7	0.17	[-0.13, 0.47]	0.27	0.0003	77	R
Pre-post intervention studies	2	**-0.37**	**[-0.45, -0.29]**	**<0.0001**	0.72	0	F
Low risk of bias	14	-0.14	[-0.40, 0.13]	0.31	<0.00001	93	R
High risk of bias	5	**-0.35**	**[-0.51, -0.20]**	**<0.0001**	0.88	0	F
Intelligence							
Observational	7	**-0.31**	**[-0.62, 0.00]**	**0.05**	<0.0001	80	R
All studies with *STH* treatment	**3**	-0.08	[-0.49, 0.32]	0.68	0.002	83	R
Experimental Studies	2	-0.19	[-0.72, 0.34]	0.48	0.002	90	R
Low risk of bias	4	-0.13	[-0.41, 0.15]	0.37	0.005	77	R
High risk of bias	5	**-0.42**	**[-0.81, -0.02]**	**0.04**	0.001	78	R
Achievement							
Observational	11	-0.12	[-0.39, 0.14]	0.36	<0.00001	93	R
All studies with *STH* treatment	**8**	-0.07	[-0.44, 0.30]	0.72	<0.00001	95	R
Experimental Intervention Studies	5	-0.27	[-0.67, 0.13]	0.18	<0.00001	95	R
Pre-post intervention studies	3	-0.30	[-0.59, 1.20]	0.51	<0.0001	95	R
Low risk of bias	8	-0.12	[-0.19, 0.43]	0.45	<0.00001	95	R
High risk of bias	6	**-0.70**	**[-1.20, -0.20]**	**0.006**	<0.00001	91	F
Attendance							
Observational	**11**	-0.23	[-0.56, 0.10]	0.17	<0.00001	98	R
Experimental Intervention Studies	5	0.03	[-0.67, 0.13]	0.18	<0.00001	95	R
Low risk of bias	4	**-0.57**	**[-1.13, -0.01]**	**0.05**	<0.00001	98	R
High risk of bias	10	-0.30	[-0.12, 0.71]	0.16	<0.00001	98	R

K: Number of studies; SMD: standard mean difference; CI; confidence interval; P_A_: P-value for association; P_B_: P-value for heterogeneity; AM: analysis model: R: Random-effects; F: Fixed-effects; I^2^: measure of variability, >50% indicates high heterogeneity; Values in **bold** indicate significant associations;

*: Includes studies with STH treatment whether pre-post intervention or experimental design.

**: Includes low ROB observational studies + Uncertain to low ROB experimental studies.

Among studies classified as low risk of bias, *STH* infection/non-treatment was associated with significant deficits in memory, learning and school attendance (SMD: -0.39 to -0.55, P <0.0001–0.03). Among studies classified as high or very high risk of bias statistical significance was achieved or maintained in the domains of learning, reaction time and intelligence (SMD: -0.28 to -1.00, P <0.001–0.03) as well as achievement (SMD: -0.70 P <0.001).

To determine whether *Schistosoma* species co-infection to contributed to observed associations between respective outcomes and *STH* infection vs. no infection among observational studies, separate pooled estimates were derived for primary studies with and without co-prevalence of *Schistosoma* species (Table 6). *STH* infection related deficits in learning (SMD = -0.32, P = 0.02) and memory (SMD = -0.32, p = 0.001) was evident in pooled analyses of primary studies without co-prevalent *Schistosoma* species infection. Among observational studies with co-prevalent schistosoma co-infection, magnitudes of association were generally stronger and pooled associations were statistically for infection related deficits in tests of learning, memory and reaction time (SMD -0.36 to -0.55, P = 0.003–0.02).

**Table 6:** Comparison of pooled effects between *STH* infection and respective outcomes with and without co-prevalent *Schistosoma* infection.

	Without *Schistosomiasis* coinfection							With *Schistosomiasis* coinfection						
		Test of Association			Test of Heterogeneity				Test of Association			Test of Heterogeneity		
Domain	K	SMD	95% CI	P_A_	P_B_	I^2^ (%)	AM	K	SMD	95% CI	P_A_	P_B_	I^2^ (%)	AM
Memory	7	**-0.32**	**[-0.51, -0.12]**	**0.001**	0.02	62	R	6	-0.18	[-0.62, 0.26]	0.42	<0.00001	93	R
Learning	6	**-0.32**	**[-0.59, -0.05]**	**0.02**	0.03	60	R	4	**-0.55**	**[-0.89, -0.21]**	**0.001**	0.0004	83	F
Reaction time	7	-0.25	[-0.60, 0.10]	0.16	<0.00001	88	R	5	**-0.55**	**[-0.92, -0.19]**	**0.003**	<0.00001	92	R
Intelligence	4	-0.42	[-1.00, 0.16]	0.16	0.0005	83	R	3	-0.21	[-0.58, 0.17]	0.28	0.007	80	R
Achievement	5	**-0.36**	**[-0.59, -0.12]**	**0.003**	0.01	77	R	5	-0.06	[-0.49, -0.61]	0.84	<0.00001	94	R
Attendance	5	-0.42	[-0.90, 0.05]	0.08	<0.00001	97	R	5	**-0.36**	**[-0.66, -0.05]**	**0.02**	0.0002	82	R

K: Number of studies; SMD: standard mean difference; CI; confidence interval; P_A_: P-value for association; P_B_: P-value for heterogeneity; AM: analysis model: R: random-effects; F: fixed-effects; I^2^: measure of variability, >50% indicates high heterogeneity; values in **bold** indicate significant associations

### Sensitivity analysis and publication bias

Table **7** presents a summary of the impact of deleting individual publications on robustness of pooled SMD in overall analyses and within strata of design, outlier study status and study quality. Of the six domains, reaction time was most robust followed by learning. In most sub-group analyses (post-outlier, observational, high risk of bias and those without *Schistosomiasis* coinfection) pooled SMDs were similar in magnitude and direction (i.e. robust) in comparison with SMDs from overall analyses. Of the 47 robust estimates, 27 (62.8%) provided statistically robust associations between *STH* infection/non-treatment and cognitive deficits or educational loss.

**Table 7:** Summary of the impact of deleting individual studies on the association between *STH* infection/non-treatment and respective outcomes in overall and sub-group analyses.

	Memory	Learning	Reaction time	Intelligence	Achievement	Attendance	A	B
Overall	Robust *	Robust *	Robust	Robust *	Robust	Robust	0	6
Post-outlier	Robust *	Robust *	Robust *	Robust	Robust *	Robust *	0	6
Observational	Robust *	Robust *	Robust *	Robust *	Robust	Robust	0	6
All studies with *STH* treatment	**[86]**	Robust *	Robust	**[49]**	**[86]**	**[67]**	3	2
Experimental intervention studies	**[49]**	Robust *	Robust	**[49]**	**[86]**	**[67]**	3	2
Pre-post intervention studies	**[17]**	**[17]**	Robust *	--------	**[17]**	--------	1	1
Low risk of bias	**[17]**	Robust *	Robust *	**[52]**	**[17]**	Robust *	2	3
High risk of bias	Robust *	Robust *	Robust	Robust *	Robust *	Robust	0	6
WSC	Robust	Robust *	Robust *	**[17]**	Robust	Robust *	1	5
WOSC	Robust *	Robust *	Robust	Robust	Robust	Robust	0	6
A	3	1	0	3	2	1		
B	6	9	10	5	6	7		

WSC: With *Schistosomiasis* coinfection; WOSC: Without *Schistosomiasis* coinfection A: Number of non-duplicate references that affected the pooled SMD; B: Number of robust comparison relative to overall findings;

* indicates statistically robust association

We found no evidence of publication bias in all six outcome domains for the overall findings and subgroups with ≥ 10 studies (Table **8**).

**Table 8:** Summary of the impact of publication bias on respective outcome domains.

		Egger Regression		Begg-Mazumdar Correlation	
	K	Intercept	P value	Kendall's τ	P value
Overall					
Memory	20	-0.29	0.87	-0.16	0.33
Learning	16	-0.32	0.84	0.03	0.86
Reaction time	20	1.59	0.25	0.18	0.26
Achievement	16	0.88	0.64	-0.23	0.21
Attendance	15	-1.70	0.74	-0.22	0.26
Observational					
Memory	14	0.64	0.66	-0.11	0.58
Learning	10	0.19	0.93	0.07	0.79
Achievement	11	1.73	0.41	-0.31	0.19
Attendance	12	-2.15	0.68	-0.03	0.89
Low Risk of Bias					
Memory	11	2.70	0.23	0.11	0.64
Reaction time	14	2.05	0.28	0.20	0.32
High Risk of Bias					
Attendance	10	5.79	0.25	0.16	0.53

K: Number of studies

## Discussion

### Principal findings and interpretations

In line with our hypothesis, we found that non-treatment for *STH* infection was consistently associated with statistically, clinically and health policy relevant deficits in five of the six domains examined. The relationships observed were generally invariant to exclusion of influential individual studies but robustness of findings varied by study design–observational, *STH* treatment studies and experimental studies, and study quality. Specifically, infection related deficits were larger in magnitude, tended to be more statistically robust and stable in direction for pooled studies of observational compared to *STH* treatment (with or without experimental) studies and for high vs. low risk of bias studies. The direction of pooled effects from observational studies with and without potential for *Schistosoma* species coinfection were similar although the magnitude of associations tended to be higher for *STH*-related deficits in settings with potential for *Schistosoma* species coinfection. These non-treatment/*STH*-infection related disadvantages in cognitive function and educational measures correspond to small to moderate deficits per the Cohen criteria [88]. Of note, these small to moderate deficits are for a highly prevalent exposure affecting millions of children in the developing world. Because reinfection is rapid in helminth endemic settings [45], many children are effectively chronically infected by these parasites and the majority of children are simultaneously polyparasitized–i.e. infected by two or more *STH* species at the same time [19]. Polyparasitic and multi-species infections may produce additive disadvantages for cognitive function and educational loss. Our finding of stronger pooled effects in *Schistosoma* prevalent settings is suggestive evidence in support of this thesis. Hence, we speculate that the modest deficits reported here may under-estimate the true magnitude of learning and memory deficits attributable to untreated *STH* infections in endemic regions.

Working memory and learning tasks involve the brain’s frontal lobe [89] which continues to develop throughout school-age years [90]. Drake et al in prior systematic reviews [18, 32] noted the lack of consistency in cognitive domains affected by helminth infections. The one exception was in *STH*-infection associated lower performance on tests of working memory [32]. Our review lends credence to that previous observation for working memory and provides new evidence of *STH* infection related deficits in learning tests. Also in line with our study hypothesis, we found that untreated *STH* infection was associated with slower reaction time. The association between *STH* infection/non-treatment and attentional processes/reaction time was evident in observational studies and persisted among intervention studies when two influential intervention studies were excluded. These suggest that *STH* infections may have an adverse effect on attention and/or cognitive speediness (i.e. reaction time).

Two findings from this meta-analyses were either not consistent with our study hypothesis or were altogether surprising. The finding that school attendance and scholastic achievement did not differ significantly in overall analyses by infection/treatment status, though similar to results from prior meta-analyses on this subject, [21, 34, 91–93] was contrary to our study hypothesis. However, we noted that the overall findings were not stable. With exclusion of influential studies and design stratified analyses we noted suggestive evidence of an adverse impact of infection/non-treatment on these outcomes among observational studies with high potential for bias but not among intervention or low risk of bias studies. Of note, scholastic achievement, as operationally defined in this study, includes children’s pass rate on standardized or teacher-made tests, the percent of children that were in appropriate class for age, child enrollment in elite versus non-elite schools, scores in the school function domain of pediatric quality-of-life inventory, change in class position after treatment, an above average versus average/below average teacher rating of scholastic performance and pass/fail rate in any kind of educational test, whether teacher administered or not. Hence, our finding of significant adverse effect of *STH* infection on this domain could have broader implications for the educational achievement in complex real-world settings. The effect on achievement likely reflect to varying degrees deficits in cognitive domains such as learning, memory and reaction time [94].

The observation of infection related deficits for tests of innate intelligence was surprising because we conceptually conceived innate intelligence as mostly a “genetically determined finite quantity” relatively insensitive to postnatal environmental assaults such as *STH* infections. Of note, this finding was robust only among observational studies and those classified as “high risk of bias”. Furthermore, the impact of infection/non-treatment for *STH* on innate intelligence became statistically insignificant with the exclusion of an influential study suggesting the need for abundant caution in the interpretation of this data. Here, we have classified neurocognitive performance assessed with variety of instruments in the primary literature into four major cognitive domains based on extant knowledge of the primary cognitive domains covered by respective tools. We supplemented as needed with content area guidance from a neuropsychologist. Innate intelligence included measures of general intelligence based on IQ tests such as the Philippine non-verbal intelligence test, Kaufmann assessment battery for children, Wechsler intelligence test for children (where overall scores and no subscales are specified). Prior reviews [32] have eloquently described the inherent challenges with neurocognitive assessments that complicates empirical efforts to understand the impact of helminth infections on disparate domains. For example, performance in respective domains likely correlate with one another to varying degrees in most individuals. Hence, it is possible that the *STH*-associated deficits in innate intelligence measures partly reflect *STH*-related effect on other domains where performance may be more sensitive to environmental factors including educational quality and health factors [95].

For this systematic review and meta-analysis, we have intentionally taken the approach of combining assessment instruments and educational measures into one of six pre-identified domains that contribute in varying degrees to the ability of children to learn, be educated and potentially their future productivity as adults. These classifications are guided by the literature and underlying theoretic constructs assessed. In spite of our best efforts, this remains an imprecise science. We provided extensive detail of our classification approach as supplemental material for critical evaluation and further refinement. In sum, the high heterogeneity [96], limited statistical power in sub-group analyses [95, 97] and the potential for residual confounding- particularly in non-randomized interventions and observational studies, [98] should lead to cautious interpretation of findings from sub-group and sensitivity analyses.

### Limitations and strengths

Our review is subject to several limitations that should be considered in the interpretation of our findings. Firstly, heterogeneity was high across studies. We analytically addressed this using random-effects model and explored the specific role of influential studies using the Galbraith plot method. Secondly, as noted earlier, our overall findings were sensitive to observational versus intervention study design and study quality. Thirdly, we are unable to fully delineate potentially *STH* species-specific differences in the domains evaluated as studies did not always distinguish between *STH* species. Additionally, across individual studies, the number of stool samples used for diagnosis of *STH* infection varied from single to multiple. Where single samples were tested, the chance of misclassifying lightly infected children increases. Thus in some studies, “the uninfected” may include an unknown number of mostly lightly infected children. Similarly, information on joint *Schistosoma* and *STH* infections as well as data on *STH* infection intensity was not consistently provided making it difficult to robustly examine potential for beyond additive adverse effects for multi-species infected children and dose-response by infection intensity. Last but not least, variations in the primary literature did not allow for subgroup analyses by the following factors–*STH* species, malnourished versus normally nourished children, type of deworming agent, and treatment strategy (mass deworming versus test and treat only infected children). These subgroup analyses–if possible, could give insight with respect to the kinds of children most likely to derive a cognitive benefit from deworming and whether mass or targeted deworming is the best approach [99] to deworming school-aged and potentially preschool children [100]. Potential for heterogeneity in the association of deworming with cognitive and educational outcomes by child nutritional status, treatment strategy, deworming agent and parasite species requires further elucidation. In light of considerable heterogeneity, varying quality of underlying studies and sensitivity to experimental versus observational study design in some outcomes, we agree with prior expressed need for appropriate caution in the interpretation of findings from synthesis of epidemiologic literature especially where associations are primarily driven by observational studies and the risk of bias in underlying studies is high or very high [101].

In spite of these limitations, our study has a number of important strengths that allow us provide additional context for understanding the influence of study design, specific influential studies and risk of bias within present literature and *STH*-associated cognitive and educational deficits. We address two issues noted by the recent Campbell review- i.e. the use of a complicated array of different cognitive tools and a lack of understanding of *STH* impact on absenteeism [101]. We enhance clarity regarding the possible impact of *STH* infection/non-treatment on educational and cognitive loss by systematically collapsing the variety of cognitive tools used within four cognitive domains and conducting specific analyses on educational loss–including scholastic achievement and absenteeism. Our approach implicitly recognizes *STH-*infections may have unequal impact in different domains despite potential over-lap in abilities tested within respective domains. It is noteworthy that in spite of major differences in our empirical approach relative to prior systematic reviews and meta-analyses on this subject, our finding of no STH-infection related deficits for most cognitive and educational outcomes in sub-group analyses restricted to experimental study designs confirms previously reported findings based on prior systematic reviews focused on RCTs only.[21, 34, 91–93] This meta-analysis allows for evaluation of consistency in primary relations investigated across key factors by including all relevant epidemiologic studies regardless of design, conducting explicit analyses to evaluate impact of influential studies, and evaluating the risk of bias in underlying studies. It has been noted that carefully implemented meta-analyses based on observational studies generally produce estimates similar to those from meta-analyses based on RCT and thus supports evidence-based medical decision-making [102, 103]. Our approach provides empirical evidence to evaluate an important health policy and yet allows allows for appropriate qualification as warranted on the basis of design, influential studies and risk of bias.

### Conclusion, remaining gaps and recommendations for future research

We provide evidence of small to modest deficit in five of six evaluated domains although there were few influential studies and variations for associations existed by study design. Prior meta-analyses of RCTs that evaluated cognitive impacts of *STH* infections had different conclusions about the cognitive and scholastic effects of *STH* infection [15, 21, 34]. Key differences in our approach–namely inclusion of all relevant epidemiologic studies and evaluation of effects within educational and cognitive domains, may partly account for the inferential difference between this and prior reviews.

Despite the empirical debate regarding the cognitive benefit of deworming for *STH*, the current ethical, clinical and health policy environments remain strongly skewed in favor of deworming for child growth, prevention of anemia and potentially avoidance of preventable cognitive deficits. Deworming efforts have expanded as a strategy to control the prevalence and intensity of infections. Ongoing operational intervention research is poised to provide valuable insight regarding the optimal approach-whether school based, community based or a combination of infection control via mass deworming will additionally examine the net benefit of bi-annual versus annual deworming [104]. Mass deworming campaigns are unlikely to interrupt infection in most settings, but infection profile is expected to shift towards low-intensity single and multi-species co-infections. Prior research has demonstrated that polyparasitism is consequential for anemia- an important determinant of cognitive deficit in children [19, 105]. Specific investigations of the cumulative impact of polyparasitic *STH* infection on cognitive and educational outcomes are lacking. Future epidemiologic studies that explicitly examine the cognitive impact of multi-species parasitic infections by number as well as by intensity of coincident infections will provide useful information to guide health policy and may inform the optimal frequency of deworming in the context of low intensity infections.

Currently, preschool-age children are not treated as part of routine deworming programs for *STH* [106], and yet, the evidence suggests that children are infected shortly after weaning and remain persistently infected throughout childhood and adolescence. The adverse impact of infection on children begins way before school age and compounds the cumulative health disadvantage associated with *STH* infection. This meta-analysis, and indeed most short-term study outcomes meta-analyzed, does not include this demographic. Recent review of investigations have demonstrated the safety and efficacy of *STH* deworming in preschool children [107]. Existence of an adverse developmental impact of *STH* infection on cognitive/educational domains would justify expanding the age-bracket of children who should be subjected to *STH* deworming. Educational and cognitive interventions will likely be more effective if initiated earlier in life for *STH*-infected children. However, more investigations among preschool children may be needed to understand the risks and potential benefits of early deworming in these children.

### Policy implications

Given the chronicity of infection during childhood and adolescence in helminth endemic regions, it is possible that any cognitive and educational loss associated with *STH* infection will not be resolved by deworming alone without prevention of reinfection and specific interventions to address the environmental and structural determinants of parasitic infections. Thus, effective future interventions are likely to be those that emphasize multi-pronged inter-sectoral strategies to holistically address challenges to child wellbeing in the developing world[108].

## Supporting information

S1 Table Psychometric tests used in the included studies classified under four cognitive and two educational domains. (DOCX)

S2 Table Proportion of single or combined helminth species. (DOCX)

S3 Table PRISMA checklist. (DOCX)

S1 Fig Forest plot of the overall results in the reaction time domain. Diamond denotes the pooled standardized mean difference (SMD). Squares indicate the SMD in each study, with square sizes directly proportional to the weight contribution (%) of each study. Horizontal lines represent 95% confidence intervals (CI). The Z test for overall effect was not significant (P >0.05) and the chi-square test indicates presence of heterogeneity (P <0.00001, I^2^ = 89%).
SD: standard deviation; CI: confidence interval; Std: standard; df: degree of freedom; I^2^: measure of variability expressed in %.
(TIF)

S2 Fig Galbraith plot analysis for the reaction time domain. The studies that lie below the -2 confidence limit are the outliers.
Log SMD: logarithm of standardized mean difference; SE: Standard error.
(TIF)

S3 Fig Effects of outlier treatment on the overall results in the reaction time domain. Diamond denotes the pooled standardized mean difference (SMD). Squares indicate the SMD in each study, with square sizes directly proportional to the weight contribution (%) of each study. Horizontal lines represent 95% confidence intervals (CI). This treatment generated gain in significance (P = 0.004) for overall effect but did not affect heterogeneity (P <0.00001, I^2^ = 78%).
SD: standard deviation; CI: confidence interval; Std: standard; df: degree of freedom; I^2^: measure of variability expressed in %.
(TIF)

S4 Fig Forest plot of the overall results in the intelligence domain. Diamond denotes the pooled standardized mean difference (SMD). Squares indicate the SMD in each study, with square sizes directly proportional to the weight contribution (%) of each study. Horizontal lines represent 95% confidence intervals (CI). The Z test for overall effect indicates significance (P < 0.05) and the chi-square test indicates that heterogeneity (P <0.00001, I^2^ = 80%).
SD: standard deviation; CI: confidence interval; Std: standard; df: degree of freedom; I^2^: measure of variability expressed in %.
(TIF)

S5 Fig Galbraith plot analysis for the intelligence domain. The study that lies below the -2 confidence limit is the outlier.
Log SMD: logarithm of standardized mean difference; SE: Standard error.
(TIF)

S6 Fig Effects of outlier treatment on the overall results in the intelligence domain. Diamond denotes the pooled standardized mean difference (SMD). Squares indicate the SMD in each study, with square sizes directly proportional to the weight contribution (%) of each study. Horizontal lines represent 95% confidence intervals (CI). This treatment resulted in loss of significance (P = 0.09) for overall effect but did not affect heterogeneity (P = 0.00001, I^2^ = 77%).
SD: standard deviation; CI: confidence interval; Std: standard; df: degree of freedom; I^2^: measure of variability expressed in %.
(TIF)

S7 Fig Forest plot of the overall results in the memory domain. Diamond denotes the pooled standardized mean difference (SMD). Squares indicate the SMD in each study, with square sizes directly proportional to the weight contribution (%) of each study. Horizontal lines represent 95% confidence intervals (CI). The Z test for overall effect is significant (P < 0.05) and the chi-square test indicates presence of heterogeneity (P <0.00001, I^2^ = 94%).
SD: standard deviation; CI: confidence interval; Std: standard; df: degree of freedom; I^2^: measure of variability expressed in %.
(TIF)

S8 Fig Effects of outlier treatment on the overall results in the memory domain. Diamond denotes the pooled standardized mean difference (SMD). Squares indicate the SMD in each study, with square sizes directly proportional to the weight contribution (%) of each study. Horizontal lines represent 95% confidence intervals (CI). This treatment did not affect significance (P < 0.05) or heterogeneity (P <0.00001, I^2^ = 84%).
SD: standard deviation; CI: confidence interval; Std: standard; df: degree of freedom; I^2^: measure of variability expressed in %.
(TIF)

S9 Fig Forest plot of the overall results in the achievement domain. Diamond denotes the pooled standardized mean difference (SMD). Squares indicate the SMD in each study, with square sizes directly proportional to the weight contribution (%) of each study. Horizontal lines represent 95% confidence intervals (CI). The Z test for overall effect is not significant (P > 0.05) and the chi-square test indicates presence of heterogeneity (P <0.00001, I^2^ = 93%).
SD: standard deviation; CI: confidence interval; Std: standard; df: degree of freedom; I^2^: measure of variability expressed in %.
(TIF)

S10 Fig Effects of outlier treatment on the overall results in the achievement domain. Diamond denotes the pooled standardized mean difference (SMD). Squares indicate the SMD in each study, with square sizes directly proportional to the weight contribution (%) of each study. Horizontal lines represent 95% confidence intervals (CI). This treatment generated gain in significance (P = 0.008) but did not affect heterogeneity (P = 0.00001, I^2^ = 90%).
SD: standard deviation; CI: confidence interval; Std: standard; df: degree of freedom; I^2^: measure of variability expressed in %.
(TIF)

S11 Fig Forest plot of the overall results in the attendance domain. Diamond denotes the pooled standardized mean difference (SMD). Squares indicate the SMD in each study, with square sizes directly proportional to the weight contribution (%) of each study. Horizontal lines represent 95% confidence intervals (CI). The Z test for overall effect is not significant (P > 0.05) and the chi-square test indicates presence of heterogeneity (P <0.00001, I^2^ = 99%).
SD: standard deviation; CI: confidence interval; Std: standard; df: degree of freedom; I^2^: measure of variability expressed in %.
(TIF)

S12 Fig Effects of outlier treatment on the overall results in the attendance domain. Diamond denotes the pooled standardized mean difference (SMD). Squares indicate the SMD in each study, with square sizes directly proportional to the weight contribution (%) of each study. Horizontal lines represent 95% confidence intervals (CI). This treatment generated gain in significance (P = 0.008) but did not affect heterogeneity (P = 0.00001, I^2^ = 98%).
SD: standard deviation; CI: confidence interval; Std: standard; df: degree of freedom; I^2^: measure of variability expressed in %.
(TIF)
