# Supplementary material for: Soil-transmitted helminth infection, loss of education and cognitive impairment in school-aged children: A systematic review and meta-analysis
Source: PLoS Negl Trop Dis. 2018 Jan 12;12(1):e0005523. doi: 10.1371/journal.pntd.0005523 (PMC5766095; doi:10.1371/journal.pntd.0005523)
Supplement: S1 Table — (DOCX) [file pntd.0005523.s001.docx]

**Table S1.** Psychometric tests used in the included studies classified under four cognitive and two educational domains

| **Cognitive Domains** | K | Combined Psychometric Tests | |
| --- | --- | --- | --- |
| *Memory* | 20 | Corsi Block | WRAML Memory |
|  |  | Verbal Fluency (measures long-term memory) | Weschler Memory Scale - Mental Control |
|  |  | WISC-Digit Span - Backwards | WISC-digit span WISC coding |
|  |  | Free Recall | Sequential / spatial memory (Boivin 1993) |
|  |  | Comprehension (Shang) | Working memory (Shang 2010) |
|  |  |  |  |
| *Learning and Executive Function* | 16 | WRAML Learning | RAVLT/ Language Delay |
|  |  | Stroop (All kinds of this test) | Comprehension - Nokes 1992 |
|  |  | Verbal analogies | Matching Familiar Figures Test |
|  |  | WiSC-III Arithmetic | Oddity Learning |
|  |  | Wisconsin Card Sorting Test | Reasoning |
|  |  | Reciprocal Motor Programs Test | French learning (Gardner 1996) |
|  |  | Spanish Vocabulary Learning | French test (Simeon 1994) |
|  |  |  |  |
| *Reaction Time* | 20 | Grooved Peg Board | Continuous performance Tests |
|  |  | Picture search | Silly Sentences |
|  |  | Coding/Code transmission Tests / Symbol | Trail Making Test / SDMT |
|  |  | Digit Cancellation Tests | Gestalt closure (Boivin 1993) |
|  |  | Visual search / recognition (Boivin 1993) | Number choice (Simeon 1995b) |
|  |  | TeaCh (CDI study) visual/spatial (Boivin 1993) | Processing speed (Shang 2010) |
|  |  |  |  |
| *Intelligence* | 9 | Raven's progressive matrix | PNIT (Philippine Non-Verbal Intelligence Test) |
|  |  | Peabody Picture Vocabulary Test - General Intelligence | DAP-III, SS |
|  |  | Kaufmann Assessment Battery for Children - (Total mental processing + nonverbal total) | FSIQ (full-scale IQ) Shang 2010 |
|  |  | WISC (if specific sub-scales are not named) | |
|  |  |  |  |
| **Educational domains** |  |  |  |
| *Achievement* | 16 | Scholastic Achievement Includes the following generally not psychometric instrument based | GMT |
|  |  | Arithmetic / Quantitative | scholastic achievement |
|  |  | School performance | General education Tamil Test |
|  |  | Reading /vocabulary/spelling | General education Math Test |
|  |  | Arithmetic Test |  |
|  |  |  |  |
| *Attendance* | 15 | Absenteeism rate |  |
|  |  | Enrollment rate |  |
|  |  |  |  |

K: number of studies; WRAML: Wide Range Assessment of Memory and Learning; WISC: Wechsler Intelligence Scale for Children; PNIT: Philippine nonverbal intelligence test; SDMT: Symbol Digit Modalities Test; RAVLT: Rey Auditory Verbal Learning Test
