# Supplementary material for: Soil-transmitted helminth infection, loss of education and cognitive impairment in school-aged children: A systematic review and meta-analysis
Source: PLoS Negl Trop Dis. 2018 Jan 12;12(1):e0005523. doi: 10.1371/journal.pntd.0005523 (PMC5766095; doi:10.1371/journal.pntd.0005523)
Supplement: S2 Table — (DOCX) [file pntd.0005523.s002.docx]

**Table S2.** Proportion of single or combined helminth species

|  | Memory | Learning | Reaction Time | Intelligence | Achievement | Attendance |
| --- | --- | --- | --- | --- | --- | --- |
| STH | 45.0 | 37.5 | 40.0 | 22.2 | 62.5 | 53.3 |
| *Trichuris* | 20.0 | 31.3 | 25.0 | 22.2 | 12.5 | 13.3 |
| Hookworm | 10.0 | 12.5 | 10.0 | 22.2 | 6.3 | 13.3 |
| *Ascaris Trichuris* | 10.0 | 12.5 | 5.0 | 22.2 | 12.5 | 20.0 |
| *Ascaris* Hookworm | 10.0 | 0 | 5.0 | 11.1 | 6.3 | 0 |
| *Ascaris* | 5.0 | 6.3 | 15.0 | 0 | 0 | 0 |
| % were obtained from the number of each species (or combined) divided by the total number of studies in each domain | | | | | | |
