# Supplementary figures and images for: Soil-transmitted helminth infection, loss of education and cognitive impairment in school-aged children: A systematic review and meta-analysis
Source: PLoS Negl Trop Dis. 2018 Jan 12;12(1):e0005523. doi: 10.1371/journal.pntd.0005523 (PMC5766095; doi:10.1371/journal.pntd.0005523)

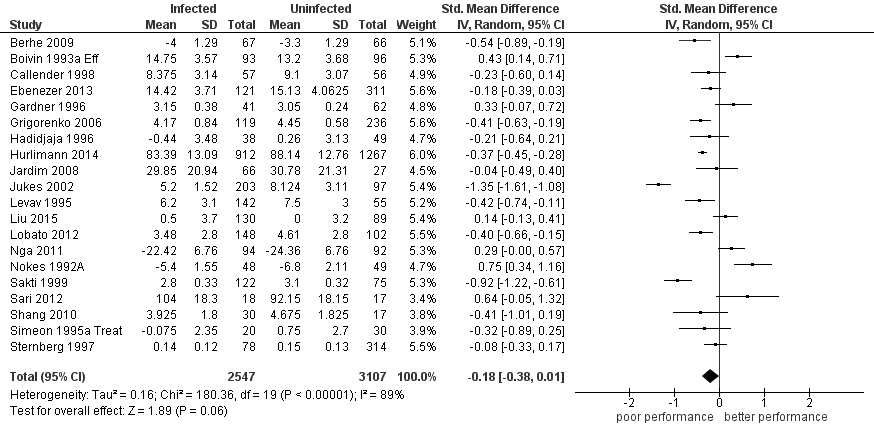

Supplement: S1 Fig — Diamond denotes the pooled standardized mean difference (SMD). Squares indicate the SMD in each study, with square sizes directly proportional to the weight contribution (%) of each study. Horizontal lines represent 95% confidence intervals (CI). The Z test for overall effect was not significant (P >0.05) and the chi-square test indicates presence of heterogeneity (P <0.00001, I2 = 89%). SD: standard deviation; CI: confidence interval; Std: standard; df: degree of freedom; I2: measure of variability expressed in %. (TIF) [file pntd.0005523.s004.tif]

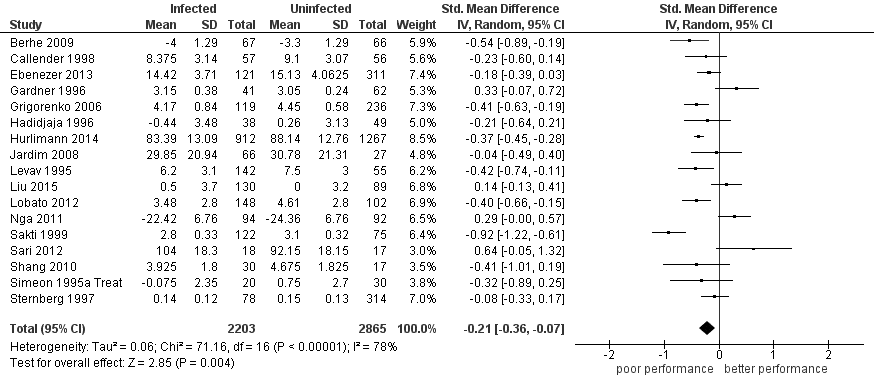

Supplement: S3 Fig — Diamond denotes the pooled standardized mean difference (SMD). Squares indicate the SMD in each study, with square sizes directly proportional to the weight contribution (%) of each study. Horizontal lines represent 95% confidence intervals (CI). This treatment generated gain in significance (P = 0.004) for overall effect but did not affect heterogeneity (P <0.00001, I2 = 78%). SD: standard deviation; CI: confidence interval; Std: standard; df: degree of freedom; I2: measure of variability expressed in %. (TIF) [file pntd.0005523.s006.tif]

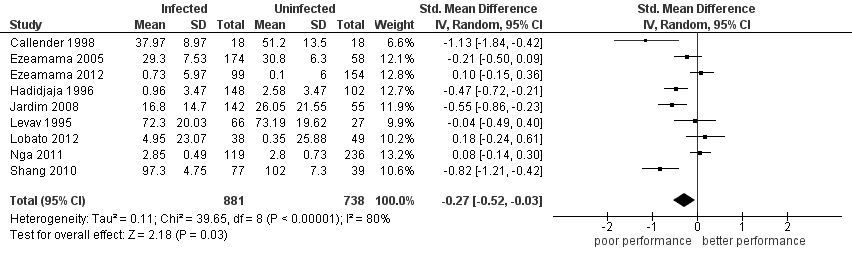

Supplement: S4 Fig — Diamond denotes the pooled standardized mean difference (SMD). Squares indicate the SMD in each study, with square sizes directly proportional to the weight contribution (%) of each study. Horizontal lines represent 95% confidence intervals (CI). The Z test for overall effect indicates significance (P < 0.05) and the chi-square test indicates that heterogeneity (P <0.00001, I2 = 80%). SD: standard deviation; CI: confidence interval; Std: standard; df: degree of freedom; I2: measure of variability expressed in %. (TIF) [file pntd.0005523.s007.tif]

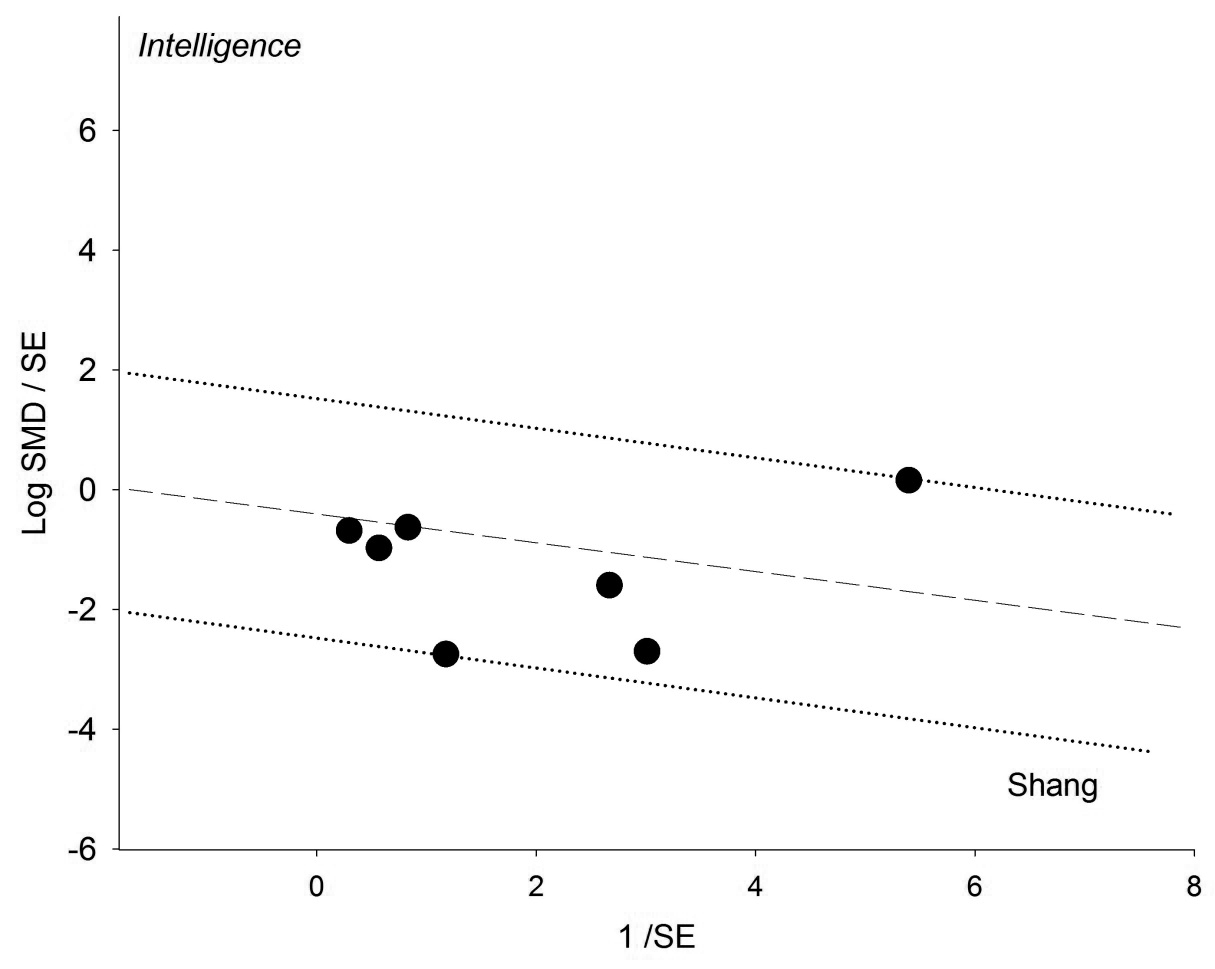

Supplement: S5 Fig — The study that lies below the -2 confidence limit is the outlier. Log SMD: logarithm of standardized mean difference; SE: Standard error. (TIF) [file pntd.0005523.s008.tif]

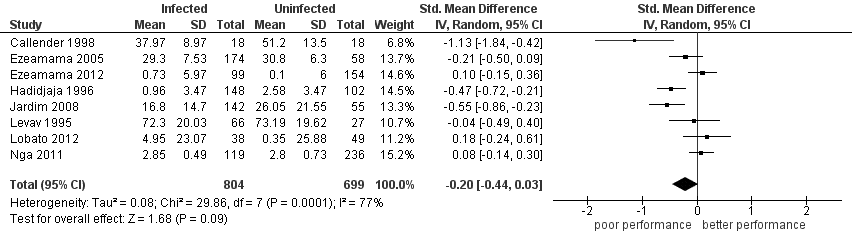

Supplement: S6 Fig — Diamond denotes the pooled standardized mean difference (SMD). Squares indicate the SMD in each study, with square sizes directly proportional to the weight contribution (%) of each study. Horizontal lines represent 95% confidence intervals (CI). This treatment resulted in loss of significance (P = 0.09) for overall effect but did not affect heterogeneity (P = 0.00001, I2 = 77%). SD: standard deviation; CI: confidence interval; Std: standard; df: degree of freedom; I2: measure of variability expressed in %. (TIF) [file pntd.0005523.s009.tif]

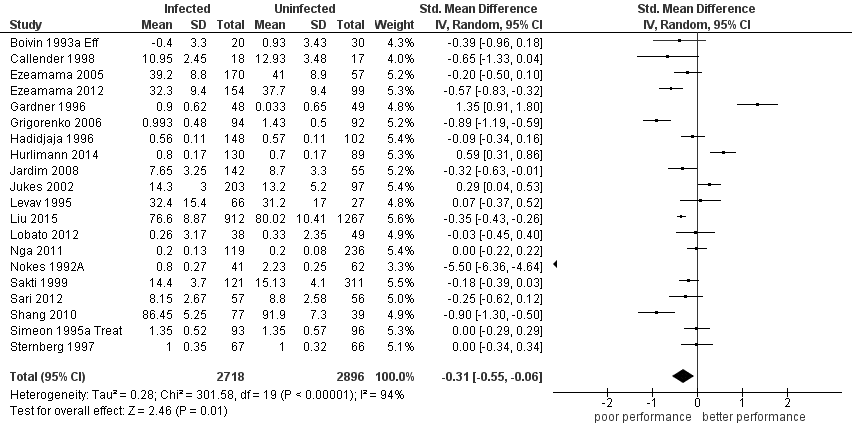

Supplement: S7 Fig — Diamond denotes the pooled standardized mean difference (SMD). Squares indicate the SMD in each study, with square sizes directly proportional to the weight contribution (%) of each study. Horizontal lines represent 95% confidence intervals (CI). The Z test for overall effect is significant (P < 0.05) and the chi-square test indicates presence of heterogeneity (P <0.00001, I2 = 94%). SD: standard deviation; CI: confidence interval; Std: standard; df: degree of freedom; I2: measure of variability expressed in %. (TIF) [file pntd.0005523.s010.tif]

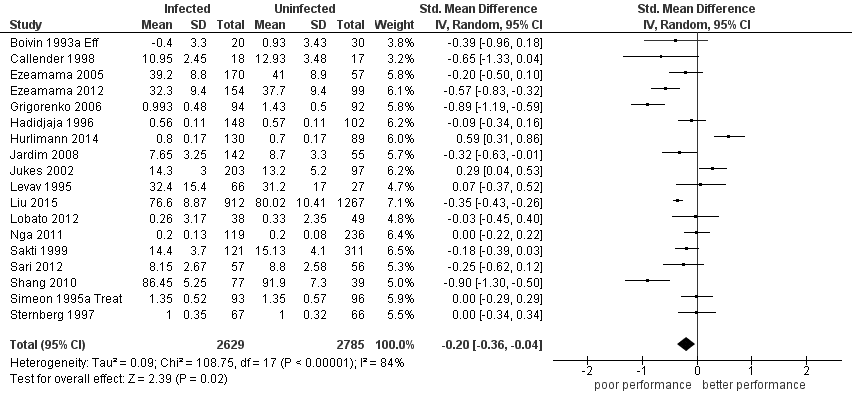

Supplement: S8 Fig — Diamond denotes the pooled standardized mean difference (SMD). Squares indicate the SMD in each study, with square sizes directly proportional to the weight contribution (%) of each study. Horizontal lines represent 95% confidence intervals (CI). This treatment did not affect significance (P < 0.05) or heterogeneity (P <0.00001, I2 = 84%). SD: standard deviation; CI: confidence interval; Std: standard; df: degree of freedom; I2: measure of variability expressed in %. (TIF) [file pntd.0005523.s011.tif]

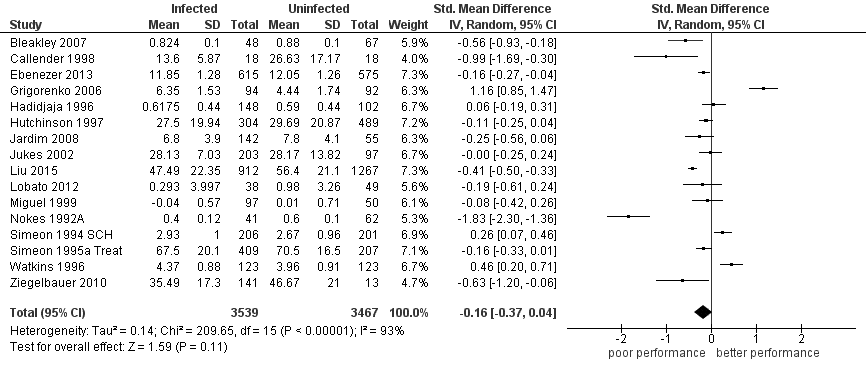

Supplement: S9 Fig — Diamond denotes the pooled standardized mean difference (SMD). Squares indicate the SMD in each study, with square sizes directly proportional to the weight contribution (%) of each study. Horizontal lines represent 95% confidence intervals (CI). The Z test for overall effect is not significant (P > 0.05) and the chi-square test indicates presence of heterogeneity (P <0.00001, I2 = 93%). SD: standard deviation; CI: confidence interval; Std: standard; df: degree of freedom; I2: measure of variability expressed in %. (TIF) [file pntd.0005523.s012.tif]

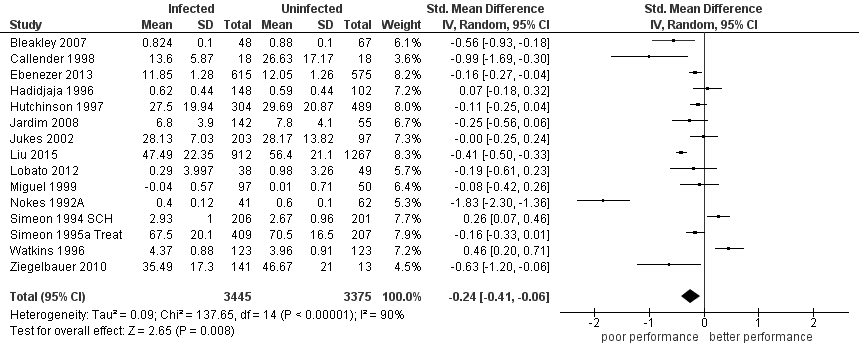

Supplement: S10 Fig — Diamond denotes the pooled standardized mean difference (SMD). Squares indicate the SMD in each study, with square sizes directly proportional to the weight contribution (%) of each study. Horizontal lines represent 95% confidence intervals (CI). This treatment generated gain in significance (P = 0.008) but did not affect heterogeneity (P = 0.00001, I2 = 90%). SD: standard deviation; CI: confidence interval; Std: standard; df: degree of freedom; I2: measure of variability expressed in %. (TIF) [file pntd.0005523.s013.tif]

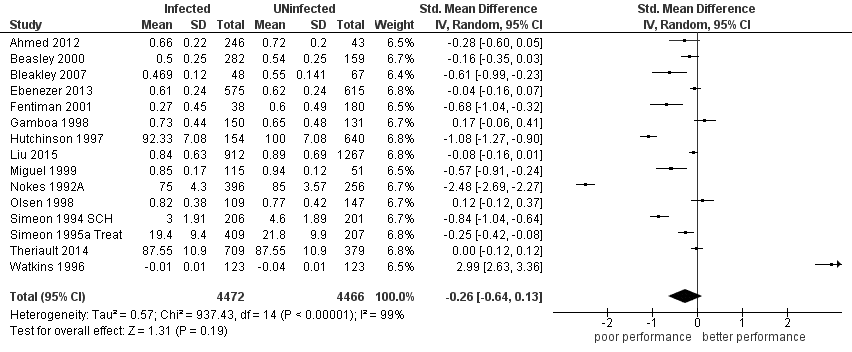

Supplement: S11 Fig — Diamond denotes the pooled standardized mean difference (SMD). Squares indicate the SMD in each study, with square sizes directly proportional to the weight contribution (%) of each study. Horizontal lines represent 95% confidence intervals (CI). The Z test for overall effect is not significant (P > 0.05) and the chi-square test indicates presence of heterogeneity (P <0.00001, I2 = 99%). SD: standard deviation; CI: confidence interval; Std: standard; df: degree of freedom; I2: measure of variability expressed in %. (TIF) [file pntd.0005523.s014.tif]

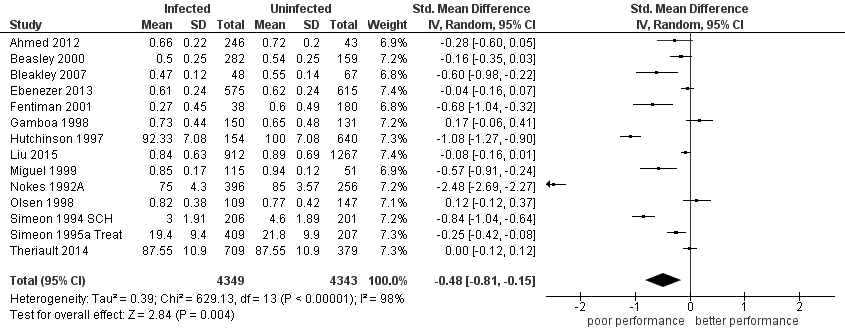

Supplement: S12 Fig — Diamond denotes the pooled standardized mean difference (SMD). Squares indicate the SMD in each study, with square sizes directly proportional to the weight contribution (%) of each study. Horizontal lines represent 95% confidence intervals (CI). This treatment generated gain in significance (P = 0.008) but did not affect heterogeneity (P = 0.00001, I2 = 98%). SD: standard deviation; CI: confidence interval; Std: standard; df: degree of freedom; I2: measure of variability expressed in %. (TIF) [file pntd.0005523.s015.tif]
